# Elabela alleviates cuproptosis and vascular calcification in vitaminD3- overloaded mice via regulation of the PPAR-γ /FDX1 signaling

**DOI:** 10.1186/s10020-024-00997-3

**Published:** 2024-11-20

**Authors:** Rui-Qiang Qi, Yu-Fei Chen, Jing Cheng, Jia-Wei Song, Yi-Hang Chen, Si-Yuan Wang, Ying Liu, Kai-Xin Yan, Xiao-Yan Liu, Jing Li, Jiu-Chang Zhong

**Affiliations:** 1 Heart Center and Beijing Key Laboratory of Hypertension, Beijing Chaoyang Hospital and Beijing Institute of Respiratory Medicine, Capital Medical University, Beijing, 100020 China; 2 Department of Cardiology, Beijing Chaoyang Hospital, Capital Medical University, Beijing, 100020 China

**Keywords:** Elabela, Cuproptosis, Peroxisome proliferators-activated receptor-γ, Vascular calcification, Vascular smooth muscle cells

## Abstract

**Background:**

Vascular calcification is a crucial pathophysiological process associated with age-related cardiovascular diseases. Elabela, a recently identified peptide, has emerged as a significant player in the regulation of cardiovascular function and homeostasis. However, the effects and underlying mechanisms of Elabela on age-related vascular calcification remain largely unexplored.

**Methods:**

In-vivo vascular calcifications of C57BL/6J mice (8-week-old) and young (8-week-old) or aged (72-week-old) SD rats were injected with vitamin D3 (VitD3) or saline, respectively. Furthermore, the VitD3-overloaded mice received Elabela (1 mg/kg/d), peroxisome proliferators-activated receptor-γ (PPAR-γ) activator Rosiglitazone (5 mg/kg/d) or copper-ionophore Elesclomol (20 mg/kg/d), respectively. As for in-vitro studies, primary rat vascular smooth muscle cells (VSMCs) were isolated from aortas and cultured for explore the role and underlying mechanism of Elabela in vascular calcification.

**Results:**

There were marked increases in FDX1 and Slc31a1 levels in both aortas and VSMCs during vascular calcification, coinciding with a rise in copper levels and a decrease in Elabela levels. Alizarin red and von-Kossa staining indicated that the administration of Elabela effectively hindered the progression of vascular cuproptosis and arterial calcification in VitD3-overloaded mice and rat arterial rings models. Moreover, Elabela significantly suppressed osteogenic differentiation and calcium deposition in VSMCs and strikingly reversed high phosphate-induced augmentation of FDX1 expression, DLAT aggregation as well as intracellular copper ion levels. More importantly, Elabela exhibited remarkable abilities to prevent mitochondrial dysfunctions in primary rat VSMCs by maintaining mitochondrial membrane potential, inhibiting mitochondrial division, reducing mitochondrial ROS production and increasing ATP levels. Interestingly, Elabela mitigated cellular senescence and production of pro-inflammatory cytokines including IL-1α, IL-1β, IL-6, IL-18 and TNF-α, respectively. Furthermore, Elabela upregulated the protein levels of PPAR-γ in VitD3-overloaded mice. Administrating PPAR-γ inhibitor GW9662 or blocking the efflux of intracellular copper abolished the protective effect of Elabela on vascular calcification by enhancing levels of FDX1, Slc31a1, Runx2, and BMP2.

**Conclusion:**

Elabela plays a crucial role in protecting against vascular cuproptosis and arterial calcification by activating the PPAR-γ /FDX1 signaling. Elabela supplementation and cuproptosis suppression serve as effective therapeutic approaches for managing vascular calcification and related cardiovascular disorders.

**Supplementary Information:**

The online version contains supplementary material available at 10.1186/s10020-024-00997-3.

## Introduction

Vascular calcification, characterized by the abnormal accumulation of calcium phosphate within the walls of vessels, results in heightened vascular stiffness and reduced compliance(Johnson et al. 2006; Lee et al. 2020a, b; Sage et al. 2010). Numerous studies have underscored the intricate and controlled features of vascular calcification, attributing a significant role to vascular smooth muscle cells (VSMCs) as pivotal players in this pathological process. In a healthy state, VSMCs showcase a contractile phenotype, marked by expression of contractile proteins like alpha-smooth muscle actin (Acta2) and smooth muscle 22 alpha (SM22α). When exposed to triggers such as dysregulations in calcium and phosphate metabolism, inflammation, and aging, VSMCs are transformed into osteoblast-like cells, characterized by upregulations in osteogenic markers like runt-related transcription factor 2 (Runx2) and bone morphogenetic protein-2 (BMP2)(Yap et al. 2021). Despite advances in comprehending the underlying mechanisms of vascular calcification, its pathophysiological complexity poses a challenge in devising effective clinical interventions.

Copper is a crucial trace element within the human body, playing vital roles in various physiological functions such as antioxidant defense and energy generation(Chen et al. 2022a, b; Tsvetkov et al. 2022; Tsang et al. 2021). However, dysregulation of copper ion metabolism has been associated with numerous cardiovascular diseases(Chen et al. 2023a, b, c, d). Cuproptosis is a novel form of programmed cell death that occurs with overloaded copper contents(Tsvetkov et al. 2022). The accumulation of excessive copper in tissues leads to its direct binding with lipoylated tricarboxylic acid cycle components, resulting in the degradation of iron-sulfur cluster-containing proteins, proteotoxic stress, and eventual cell death. The key regulator, Ferredoxin 1 (FDX1), is involved in cuproptosis and protein lipoylation, encoding a reductase responsible for converting copper ions to its more toxic form, which binds to lipoylated proteins, inducing proteotoxic stress(Tsvetkov et al. 2022; Chen et al. 2023a, b, c, d). Slc31a1, also known as the copper transporter 1, plays a critical role in cellular copper homeostasis by facilitating the uptake of copper ions into cells(Das et al. 2022). Given that Slc31a1 regulates intracellular copper levels, its expression and activity directly influence the susceptibility of cells to cuproptosis. Dihydrolipoamide acetyltransferase (DLAT) is a crucial component of the pyruvate dehydrogenase complex, playing a significant role in cellular metabolism by facilitating the conversion of pyruvate to acetyl-CoA(Tsvetkov et al. 2022; Das et al. 2022). Recent research has illuminated the involvement of DLAT in copper-induced cell death, known as cuproptosis(Tsvetkov et al. 2022; Chen et al. 2024). Elevated levels of copper can lead to the binding of copper ions to DLAT, resulting in the disruption of its enzymatic function and subsequent mitochondrial dysfunction(Chen et al. 2022a, b). This disruption triggers a cascade of oxidative stress and energy depletion, ultimately leading to cuproptosis.

Disorders in copper homeostasis are implicated in various vascular diseases, such as atherosclerosis, vascular remodeling, and diabetic endothelial dysfunction(Chen et al. 2023a, b, c, d; Das et al. 2022; Zhong et al. 2024). However, the mechanisms between cuproptosis with vascular calcification remain largely unknown. Furthermore, dysregulated metal ion metabolism has been linked to the progression of cellular senescence(Wang et al. 2020; Tian et al. 2022). As cells age, their capacity to regulate calcium and phosphate homeostasis decreases, making them more susceptible to mineral deposition and ossification(Lee et al. 2020a, b; Sutton et al. 2023). Cellular senescence has been exhibited to heighten the osteogenic differentiation of VSMCs, further contributing to vascular calcification(Sutton et al. 2023). Vascular calcification and the osteo/chondrogenic conversion of VSMCs are distinctive features of vascular aging, which is associated with various age-related conditions such as Parkinson’s disease, hypertension, and diabetes(Sutton et al. 2023; Li et al. 2024). Abnormalities in copper metabolism contributes to the development and progression of Alzheimer’s disease, as well as accelerating the aging process, highlighting a correlation between aging and copper metabolism(Wang et al. 2020). Studies have found that serum copper levels remain elevated in elderly individuals(Li et al. 2019), and alterations in cellular copper content can greatly impact the process of vascular aging and calcification(Chen et al. 2023a, b, c, d). Nonetheless, the specific mechanisms of vascular calcification and potential therapeutic targets remain incompletely understood in the context of vascular aging.

Elabela/APELA (Ela) is a 54-amino acid peptide identified as a novel ligand for the apelin receptor (Chapman et al. 2023; Monastero et al. 2023). The APJ receptor, a G-protein-coupled receptor, is highly expressed in the cardiovascular system(Chapman et al. 2023). Numerous studies have suggested that targeting Elabela and APJ could be a promising therapeutic strategy for cardiovascular disorders(Chapman et al. 2023; Peng et al. 2024; Xi et al. 2023; Chen et al. 2022a, b; Ye et al. 2022; Yang et al. 2017; Hu et al. 2022). Our research group has uncovered the diverse cardiovascular protective effects of the Elabela-APJ system in recent years(Zhang et al. 2022, 2023a, b; Song et al. 2020). Intriguingly, our previously reported that Elabela alleviates hypertension-mediated cardiac ferroptosis, myocardial fibrosis and hypertrophy by modulating the IL-6/STAT3/GPX4 signaling pathway(Zhang et al. 2022). The Elabela-APJ signaling axis has been shown to have a protective role following myocardial infarction by promoting angiogenesis (Peng et al. 2024; Xi et al. 2023). In addition, chronic infusion of Elabela alleviates pressure-overload-induced vascular remodeling via anti-inflammatory, anti-oxidative and anti-proliferative properties(Ye et al. 2022). Elabela treatment also significantly reduced monocrotaline-induced pulmonary vascular remodeling and associated right heart remodeling(Yang et al. 2017; Hu et al. 2022). Moreover, it has been reported that Elabela is implicated in different forms of cell death, including apoptosis(Xi et al. 2023; Wang et al. 2022a, b), ferroptosis(Zhang et al. 2022; Xu et al. 2023) and pyroptosis(Chen et al. 2023a, b, c, d). However, the precise role of Elabela in cuproptosis and vascular calcification remains largely unclear. In this work, we explored the roles and underlying molecular mechanisms of Elabela in cuproptosis and vascular calcification by using VitD3-induecd vascular calcification, arterial rings and VSMCs calcification models.

## Materials and methods

### Animal experiments

All animal experiments were approved by the Institutional Animal Care Committee at Beijing Chaoyang Hospital, Capital Medical University, Beijing, China and performed in accordance with the US National Institutes of Health Guide for the Care and Use of Laboratory Animals. Vitamin D3 (VitD3, MedChemExpress, 5.5 × 10^5^ IU/kg subcutaneous injection) was injected once a day for three times to induce calcification models in young (8-week-old) and aged (72-week-old) SD rats, as well as C57BL/6J mice (8-week-old) as previous described(Chen et al. 2023a, b, c, d; Wang et al. 2022a, b). In some experiments, VitD3-overloaded mice were received with Elabela (1 mg/kg/d, intraperitoneal injection, MedChemExpress), PPAR-γ activator Rosiglitazone (5 mg/kg/d, intraperitoneal injection, MedChemExpress) or Elesclomol (20 mg/kg/d, subcutaneous injection, Topscience), respectively. All animals were kept under standard conditions, including temperature range of 22–25 °C and12-hour light/dark cycle. After treatment for 7 days, the mice were euthanized, then the aortas were collected for further analysis.

### Arterial ring organ culture

As previous described(Chen et al. 2023a, b, c, d), two-month-old male SD rats were euthanized, and thoracic aortas were dissected under sterile conditions. The arteries were then cut into 5–6 mm rings for consistency. Subsequently, the vascular rings were subjected to treatment with growth medium (GM, DMEM/F12 medium) or calcifying medium (CM, DMEM/F12 medium containing 10 mM β-GP and 3 mM CaCl_2_), with or without Elabela (1 µM), Elesclomol (50 nM), or GW9662 (5 µmol) in the presence of CM in a 37 ℃ incubator with 5% CO_2_. To induce vascular calcification, the arterial rings were immersed in CM supplemented with 10 mM of β-glycerophosphate (β-GP, Sigma-Aldrich, St. Louis, MO, USA) and 3 mM CaCl_2_ (Sigma-Aldrich, St. Louis, MO, USA) for a duration of 7 days.

### Cell culture and treatment

Primary rat VSMCs were isolated from aortas by using the explant method(Chen et al. 2023a, b, c, d). Following euthanasia, the thoracic aortas were isolated; then the adventitia and intima were removed. The aortas were then cut into small pieces to create aortic explants, which were cultured in DMEM/F12 medium (Thermo Fisher Scientific, Waltham, MA, USA) supplemented with 10% fetal bovine serum (FBS, Thermo Fisher Scientific, Waltham, MA, USA), 100 U/mL penicillin, and 100 µg/mL streptomycin (Pricella, Wuhan, China). VSMCs were passaged when they reached approximately 80% confluence. To induce osteogenic differentiation and cell calcification, VSMCs were treated with CM (containing 10 mM β-GP and 3 mM CaCl_2_) for 7 days. Elabela was administered at a concentration of 1 µM in the presence of CM. The GM and CM medium were refreshed every 2 days. In some experiments, Elesclomol (50 nM), GW9662 (5 µmol) and H_2_O_2_ (100 µM) were used to treat rat VSMCs.

### Small interfering RNA (siRNA) transfection

The siRNA used in this study was obtained from GenePharma Co. Ltd. (Shanghai, China). When VSMCs reached 80% confluence, transfection was carried out with 100nM ATP7a siRNA or scrambled siRNA (si-NC) using Lipofectamine RNAiMAX Transfection Reagent (Thermo Fisher Scientific, Waltham, MA, USA) in accordance with the manufacturer’s instructions. Following 24 h of transfection, the medium was changed, and additional experiments were conducted. The protein level of ATP7a in VSMCs was evaluated through Western Blot analysis.

### Western blot analysis

Total protein was extracted from VSMCs or arterial tissues using radio immunoprecipitation assay (RIPA) lysis buffer (Solarbio, Beijing, China) containing proteinase (Roche, Mannheim, BW, Germany). The protein concentration was determined using a BCA protein assay kit (Thermo Fisher Scientific, Waltham, MA, USA). Next, protein was separated by sodium dodecyl sulfate polyacrylamide gel electrophoresis (SDS-PAGE) and transferred to a polyvinylidene fluoride (PVDF) membrane (Merck Millipore, Billerica, MA, USA). After blocking with 5% milk for 1 h at room temperature, the membranes were incubated overnight at 4 °C with the specified primary antibodies as detailed in the supplementary materials. Subsequently, the membranes were washed three times with TBST and incubated with secondary anti-rabbit or anti-mouse HRP-conjugated antibodies for 2 h at room temperature. Antibody binding was visualized using ECL detection reagent (Bio-Rad, Hercules, CA, USA). Protein bands were analyzed with Image J software and normalized to GAPDH or Tubulin expression.

### Quantitative real-time polymerase chain reaction (qRt-PCR)

Total RNA was extracted from tissues and VSMCs by using TRIzol reagent (Invitrogen, Waltham, MA, USA). Subsequently, the RNA was quantified and reverse-transcribed using a PrimeScript RT reagent kit (TaKaRa, Tokyo, Japan) following the manufacturer’s instructions. Gene expression levels were measured via quantitative PCR using Synergy Brands (SYBR) Green mixture in a 7500 FAST Real-Time PCR System (Applied Biosystems, Foster City, CA, USA). The primers utilized in this study were synthesized by Sangon Biotech (Shanghai, China), and the sequences can be found in the supplementary materials (Supplementary Table 1).

### Immunofluorescence and immunohistochemistry staining

Aortic tissues and cultured VSMCs were collected at specified time points. For tissue staining, arteries were fixed in 4% paraformaldehyde (Solarbio, Beijing, China) overnight and then embedded in paraffin. Sections of 4–6 μm thickness were prepared for further staining. The paraffin-embedded sections were deparaffinized, and antigen retrieval was carried out using EDTA Antigen Retrieval Solution. After blocking with 5% bovine serum albumin (BSA, Solarbio, Beijing, China) for 30 min at room temperature, the sections were incubated with primary antibodies diluted in immunostaining primary antibody dilution solution overnight at 4 °C. Subsequently, FITC-labeled or Alexa Fluor 647 labeled secondary antibodies were applied for 1 h at room temperature. The coverslips were mounted on glass slides using a mounting medium (Beyotime, Shanghai, China) containing DAPI for counterstaining of the nuclei. Imaging was performed using an Olympus IX73 fluorescence microscope. For immunohistochemistry, the endogenous peroxidase activity in tissues was blocked with 3% H_2_O_2_ and then blocked with 5% BSA. The samples were incubated with primary antibodies overnight, followed by staining with an HRP-labeled secondary antibody at room temperature for 45 min, and visualization with DAB. Finally, the sections were counterstained with hematoxylin solution and observed using a microscope (Leica, Wetzlar, Germany).

For VSMCs, the cells were washed with 1× PBS, fixed in 4% paraformaldehyde for 30 min, permeabilized with 0.3% Triton-X for 20 min at room temperature, blocked with 5% BSA for 30 min, and then incubated with the primary antibody overnight at 4 °C. After washing, the VSMCs were incubated with Alexa Fluor secondary antibodies for 1 h at room temperature in the dark. The cell nuclei were counterstained with DAPI, and images were captured using a fluorescence microscope.

### Alizarin red staining

As previously described in the literature(Li et al. 2024; Chen et al. 2023a, b, c, d), alizarin red staining was utilized to evaluate the calcification of VSMCs. Briefly, VSMCs were fixed in 4% paraformaldehyde for 10 min and then stained with a 0.2% alizarin red solution (Solarbio, Beijing, China) for 3 min. As for vascular tissues, sections were cut and mounted on glass slides. The sections were then incubated with 2% Alizarin Red S solution for 30 min at room temperature, allowing the dye to bind specifically to calcium deposits. After thorough washing with distilled water, the slides were counterstained with hematoxylin. Finally, the stained sections were examined under a light microscope, and images were captured for quantitative analysis of calcification.

### Von-Kossa staining

Arterial samples were fixed in 10% formalin solution, embedded in paraffin, cut into sections, and then stained with 5% silver nitrate (Servicebio, Wuhan, China) followed by exposure to ultraviolet light. The stained sections were examined under a light microscope, and images were captured for quantitative analysis.

### Calcium content assay

The measurement of calcium levels in VSMCs and arterial tissues was conducted using Calcium Test Kits (Nanjing Jiancheng Bioengineering, Nanjing, China) following the guidelines outlined by the manufacturer. Initially, the cells or tissues underwent homogenization and centrifugation for the collection of the supernatants. These supernatants were then treated with methyl thymol blue solution for a duration of 5 min at ambient temperature, with subsequent measurement of optical density at 610 nm. The quantification of protein concentration was achieved through the utilization of a BCA assay kit. Normalization of calcium content was performed in relation to protein concentrations and reported as µg/mg of protein.

### Alkaline phosphatase (ALP) activity assay

The assessment of ALP activity was carried out using an ALP assay kit (Nanjing Jiancheng Bioengineering, Nanjing, China) as per the instructions. Arteries were homogenized, resulting in the isolation of supernatants through the process of centrifugation. The samples were then added p-nitrophenyl phosphate (p-NPP) substrate and were incubated for a period of 10 min at 37 ℃. The reaction was halted with 3 mol/l NaOH, following which the absorbance was determined at 405 nm. The quantification of protein concentration was ascertained utilizing a BCA assay kit. Subsequently, the outcomes were adjusted based on the protein levels, with the ALP activity being calculated in units per milligram of protein.

### Copper content detection

Copper Microplate Assay Kit (Absin, Shanghai, China) was used to measure the copper content in vascular tissues and VSMCs. In brief, the supernatant of sample homogenate was mixed with the assay reagent and incubated at room temperature for 15 min. The absorbance of the samples at 605 nm was then measured using an Enzyme-linked immunosorbent assay reader. The quantification of protein concentration was ascertained utilizing a BCA assay kit. Subsequently, normalization of copper content was performed in relation to protein concentrations.

### Coppersensor-1 staining

To assess intracellular copper levels, VSMCs were cultured in different medium supplemented with 1 µM copper. After 24 h, cells were incubated with 5 µM Coppersensor-1, a synthetic fluorophore for cell copper imaging, for 30 min at 37 °C. Following incubation, cells were washed and analyzed using fluorescence microscopy.

### Detection of mitochondrial function

Mitochondrial damage in VSMCs were assessed by measuring the mitochondrial membrane potential using the JC-1 staining assay kit (Beyotime, Shanghai, China). VSMCs were treated with the JC-1 working solution for 30 min as per the manufacturer’s instructions. Fluorescence microscopy was utilized to capture images, and the Red/Green fluorescence intensity ratio was analyzed to assess potential mitochondrial damage. Furthermore, mitochondrial permeability transition pore opening was measured using the Mitochondrial Permeability Transition Pore (MPTP) Assay Kit (Beyotime, Shanghai, China). The assay was conducted according to the manufacturer’s guidelines. After treatment with stimuli, VSMCs were stained with a fluorescence probe (Calcein AM) provided in the kit, and images were captured using a fluorescence microscope. MitoSOX Red mitochondrial superoxide indicator (Beyotime, Shanghai, China) was used to detect the levels of superoxide in mitochondria. MitoTracker Deep Red FM (Beyotime, Shanghai, China) staining was utilized to investigate morphological alteration of mitochondria.

### Measurement of ATP levels

According to the manufacturer’s protocol, an ATP Assay Kit (Nanjing Jiancheng Bioengineering, Nanjing, China) was used to measure the ATP level in cells. VSMCs were harvested and lysed in a buffer to release intracellular ATP. Then, the lysates were then mixed with working reagents, following which the absorbance was determined at 636 nm. Subsequently, absorbance was measured using enzyme labelling equipment and ATP concentration was calculated based on a standard curve generated with known concentrations of ATP.

### Assessment of β-galactosidase (β-gal) activity

To analyze senescence, the β-gal assay (Beyotime, Shanghai, China) was employed. VSMCs were cultured in 6-well plates and subjected to H_2_O_2_ treatment to induce senescence. After rinsing with PBS, the VSMCs were fixed at room temperature for 30 min and then incubated wit β-gal staining solution overnight at 37 °C. The percentage of senescent cells was determined by the ratio of blue-stained cells to the total cell count.

### Measurement of NAD^+^ levels

The NAD^+^ levels was determined by using an assay kit (Beyotime, Shanghai, China). Cellular samples were lysed with the provided extraction buffer, mixed with ethanol dehydrogenase working solution, and incubated at 37 °C for 10 min. The enzymatic reaction was stopped, and the absorbance was read at 450 nm. Subsequently, NAD^+^ levels were normalized to the total protein content.

### Statistical analysis

Statistical analyses were conducted using Graphpad Prism 9. Data was presented as mean ± SD. Group differences were compared using Student’s t-test for two-group comparisons and one-way analysis of variance (ANOVA) for multiple group comparisons. Statistical significance was considered at a P value below 0.05.

## Results

### Elabela expression is decreased in vascular calcification accompanied with augmentation of cuproptosis

**Fig. 1:**
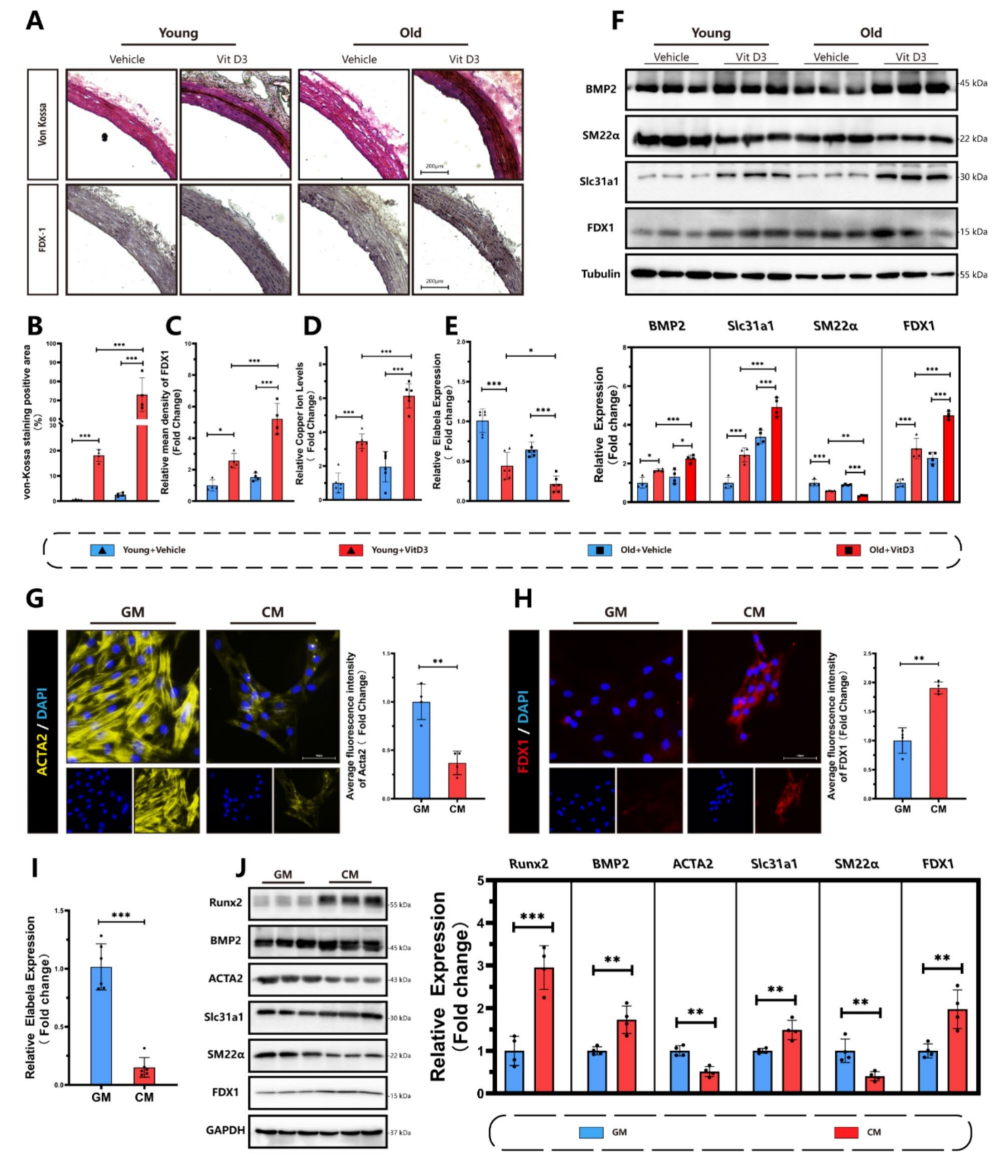
Downregulation of Elabela and activation of cuproptosis in aortic tissues and VSMCs in response to vascular calcification. **(A-C)** Representative image and quantification of von-Kossa staining (**A, B**) and immunohistochemical staining of FDX1 (A, C) in rat aortas. Scale bar = 200 μm, *n* = 4. **(D)** Measurement of copper content in vascular tissues, *n* = 6. **(E)** qRT-PCR analysis of Elabela expression in aorta of VitD3 overload mice, *n* = 6. **(F)** Representative Western blots images and quantification of Slc31a1, FDX1, BMP2 and SM22α in aortic tissues in both young and aging rats treated with or without VitD3, *n* = 4 per group. **(G**,**H)** Representative immunofluorescence and quantitative analyses of Acta2 (**G**) (Yellow), FDX1 (**H**) (Red) and DAPI (Blue) in VSMCs. Scale bar = 100 μm, *n* = 4. **(I)** qRT-PCR analysis of Elabela expression in VSMC treated with GM or CM, *n* = 6 per group. **(J)** Representative western blots images and quantification of Slc31a1, FDX1, BMP2, Acta2, Runx2 and SM22α expressions in VSMCs, *n* = 4 per group. Con, Control; VitD3, Vitamin D3; GM, growth medium; CM, calcifying medium; Ela, Elabela; Runx2, runt-related transcription factor 2; BMP2, bone morphogenetic protein-2; Acta2, alpha-smooth muscle actin; SM22α, smooth muscle 22 alpha; FDX1, ferredoxin 1; Slc31a1, solute carrier family 31 member 1. * *P* < 0.05, ** *P* < 0.01, *** *P* < 0.001

We first explored the relationship between Elabela, cuproptosis and vascular calcification in VitD3-overloaded calcification model. Von-Kossa staining and immunohistochemistry staining for FDX1 were conducted to confirm vascular calcification in both young and aging rats (Fig. 1. A-C). Following the injection of VitD3, there was a substantial increase in the protein levels of FDX1 and tissue copper content in aortas (Fig. 1. D). Interestingly, a significant decrease in mRNA levels of Elabela was observed during this process (Fig. 1. E). The ELISA assay also revealed that serum Elabela levels decreased in mice injected with VitD3 (Fig S1). Western blots (Fig. 1. F) and qRt-PCR (Fig S2) further demonstrated that VitD3 treatment upregulated the expression of FDX1 and Slc31a1 in the rats’ aortic tissues, suggesting the activation of cuproptosis in the progression of vascular calcification. Bioinformatics analysis of GSE146638 (Fig S2) also indicated an upregulation of cuproptosis-associated markers in rats, including FDX1 and Slc31a1, in Vitamin D3-induced vascular calcification. Notably, the activation of the cuproptosis signal and augmentation of vascular calcification were observed in aging rats injected with VitD3 compared to their younger counterparts (Fig. 1. A-D). Additionally, the mRNA level of Elabela was further decreased in aging rats (Fig. 1. E). To validate these findings in vitro, VSMCs were induced by high phosphate stimulation. Immunofluorescence results exhibited a decrease in the contractile marker Acta2 expression (Fig. 1. G) in primary rat VSMCs, while the fluorescence intensity of FXD1 (Fig. 1. H) was significantly increased. Similarly, there was a notable increase in the expression of osteogenic markers, including Runx2 and BMP2 (Fig. 1. J), in VSMCs cultured in CM compared to those in GM conditions. Furthermore, the expression of FDX1 and Slc31a1 in VSMCs were significantly increased in the calcification medium group (Fig. 1. J). In addition, mRNA levels of Elabela (Fig. 1. I) were significantly downregulated in the CM-treated VSMCs. Taking together, these findings suggest an activation of cuproptosis coinciding with increases in FDX1 and Slc31a1 levels and a decrease in Elabela expression during vascular calcification.

### Elabela attenuates vascular calcification and cuproptosis in VitD3-overloaded mice

**Fig. 2:**
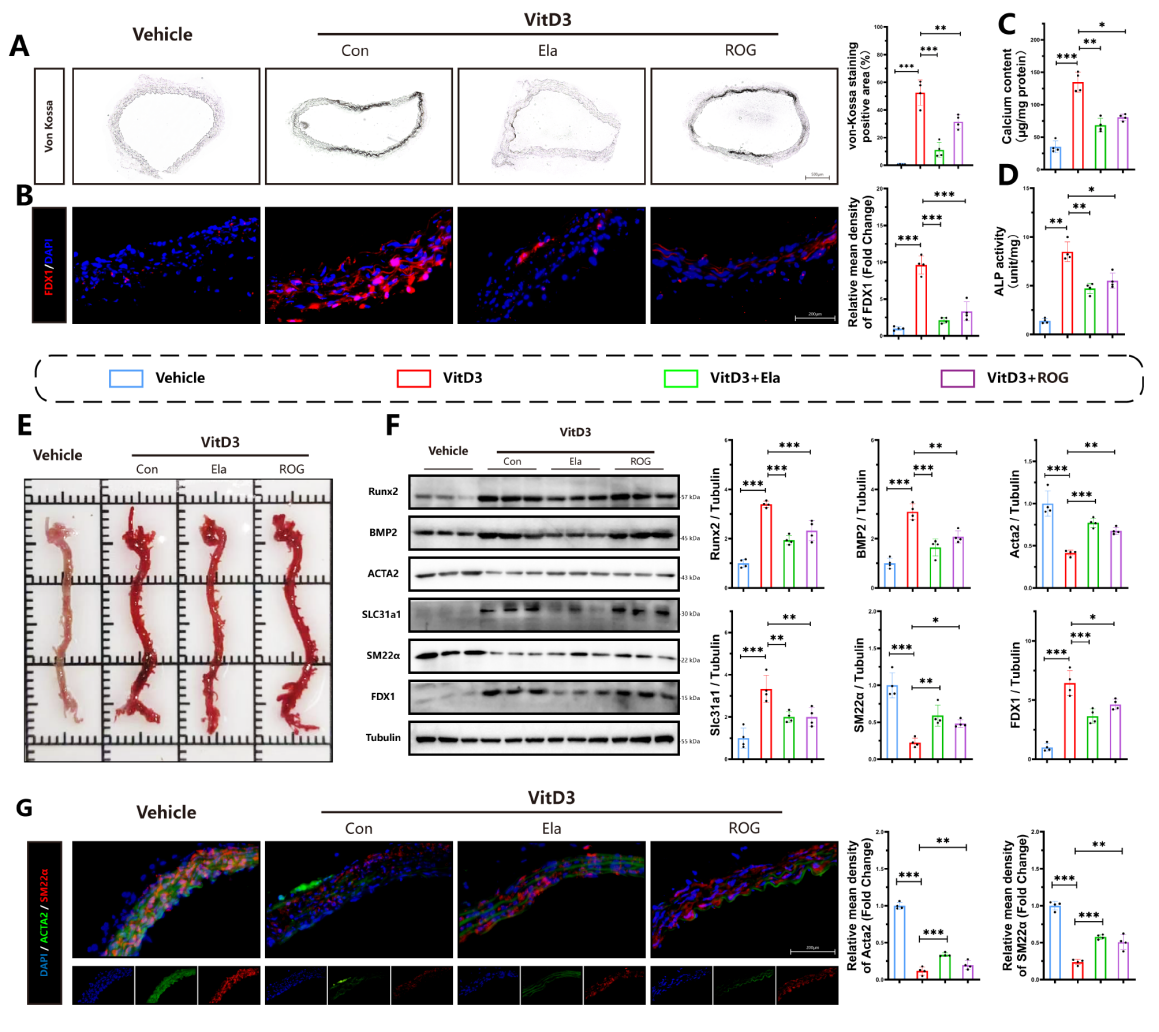
Elabela attenuates vascular calcification and cuproptosis in VitD3 overloaded mice. **(A)** Representative image and quantification of von-Kossa staining in mice injected with vitamin D3. Scale bar = 200 µm, n = 4. **(B)** Immunofluorescence staining of FDX1 (Red). Scale bar = 200 µm, n = 4. **(C)** Quantification of calcium content in vascular tissues, n = 4. **(D)** Quantification of alkaline phosphatase activity in vascular tissues, n = 4. **(E)** Representative Alizarin Red S staining images of the whole aortas from mice injected with VitD3. **(F)** Representative western blots and quantification of Slc31a1, FDX1, BMP2, Acta2, Runx2 and SM22α in mice aorta (n = 4 per group). **(G)** Immunofluorescence staining was performed for Acta2 (Green), SM22α(Red) and DAPI (Blue) in mice aorta. Scale bar = 200 µm, n = 4. Con, Control; VitD3, Vitamin D3; Ela, Elabela; ROG, rosiglitazone; Runx2, runt-related transcription factor 2; BMP2, bone morphogenetic protein-2; Acta2, alpha-smooth muscle actin; SM22α, smooth muscle 22 alpha; FDX1, ferredoxin 1; Slc31a1, solute carrier family 31 member 1; DAPI, 4’,6-diamidino-2-phenylindole. *P < 0.05, **P < 0.01, ***P < 0.001

To determine related signaling in vascular calcification, we treated VitD3-overloaded mice with Elabela or ROG, an activator of PPAR-γ. Von-Kossa staining of aortic sections (Fig. 2A) and Alizarin red staining of total aortas (Fig. 2E) revealed that aortic calcification was induced by VitD3 while attenuated by Elabela or ROG, respectively. Similarly, vascular tissue calcium content assays (Fig. 2C) and ALP activity levels (Fig. 2D) further confirmed that Elabela could inhibit calcification-related phenotypes. Additionally, administration of Elabela or ROG resulted in increased protein expression of contractile markers (Acta2 and SM22α) and decreased expression of osteogenic markers (Runx2 and BMP2) in mice with VitD3 overload (Fig. 2. F). Immunofluorescence results (Fig. 2. G) further supported these findings, demonstrating that Elabela treatment could reverse the VitD3-mediated decreases in aortic levels of Acta2 and SM22α in mice. Of note, VitD3 overload upregulated expressions of FDX1, Slc31a1 (Fig. 2. B) and copper levels (Fig S3) in aortic calcification, which was significantly abolished by Elabela supplementation, suggesting that supplementation with Elabela can alleviate aortic calcification and cuproptosis in mice with VitD3 overload.

### Elabela reduces the aggravation of vascular calcification by inhibiting cuproptosis

**Fig. 3:**
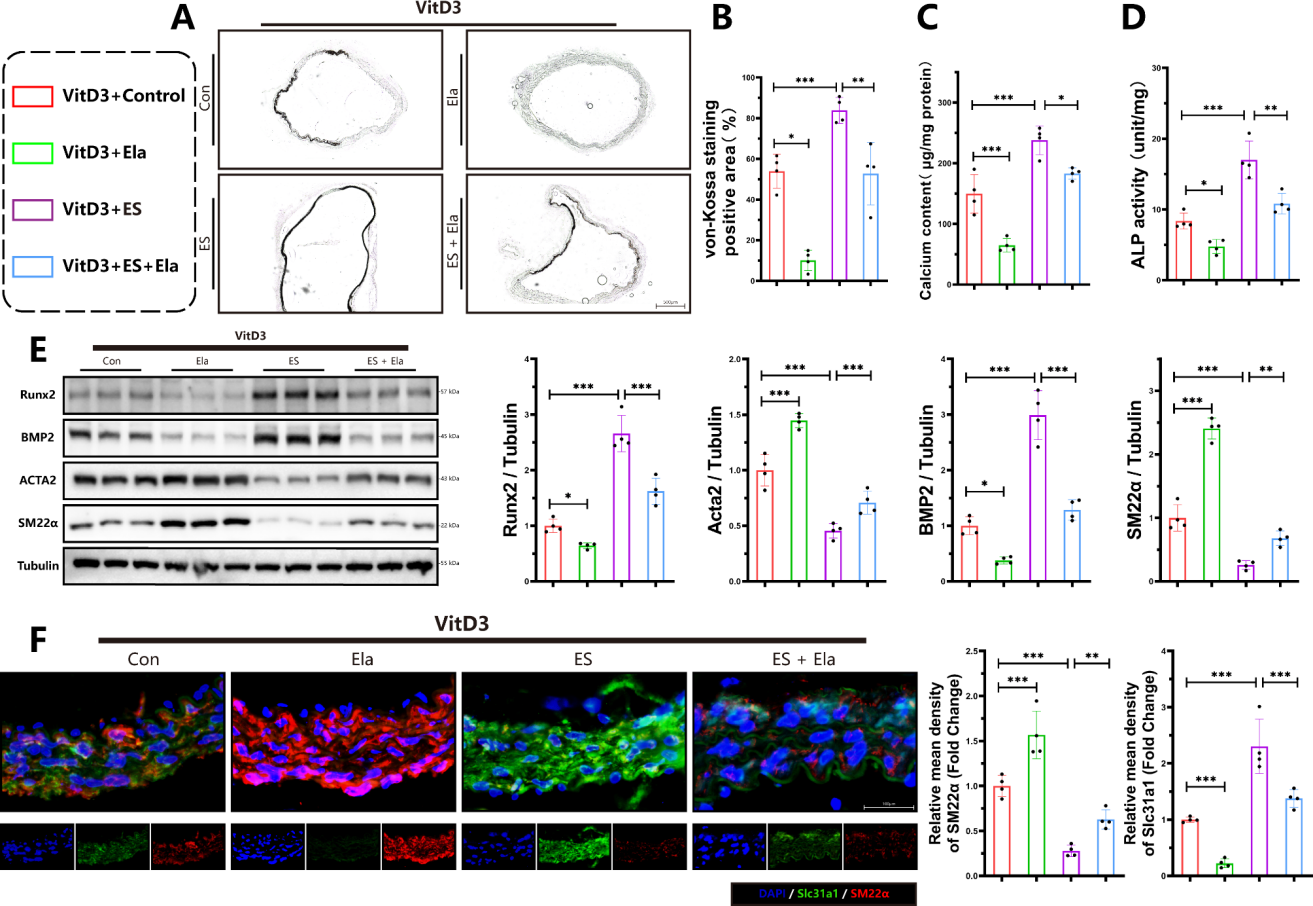
Elabela impedes exacerbation of vascular calcification induced by cuproptosis. **(A**,**B)** Calcium deposition in mice aortas was stained with von-Kossa staining and quantified by Image J software. Scale bars = 200 µm, n = 4 per group. **(C)** Quantification of calcium content in vascular tissues of VitD3-treated mice, n = 4. **(D)** Quantification of alkaline phosphatase activity in vascular tissues of VitD3-treated mice, n = 4. **(E)** Representative western blot images and quantitative analysis of osteogenic (BMP2 and Runx2) and contractile (Acta2 and SM22α) property factors expression in arteries of VitD3-treated mice. **(F)** Immunofluorescence staining was performed for Slc31a1 (Green), SM22α(Red) and DAPI (Blue) in aortas of VitD3-treated mice. Scale bar = 200 µm, n = 4 per group. Con, Control; VitD3, Vitamin D3; Ela, Elabela; ES, elesclomol; Runx2, runt-related transcription factor 2; BMP2, bone morphogenetic protein-2; Acta2, alpha-smooth muscle actin; SM22α, smooth muscle 22 alpha; Slc31a1, solute carrier family 31 member 1; DAPI, 4’,6-diamidino-2-phenylindole. *P < 0.05, **P < 0.01, ***P < 0.001

**Fig. 4:**
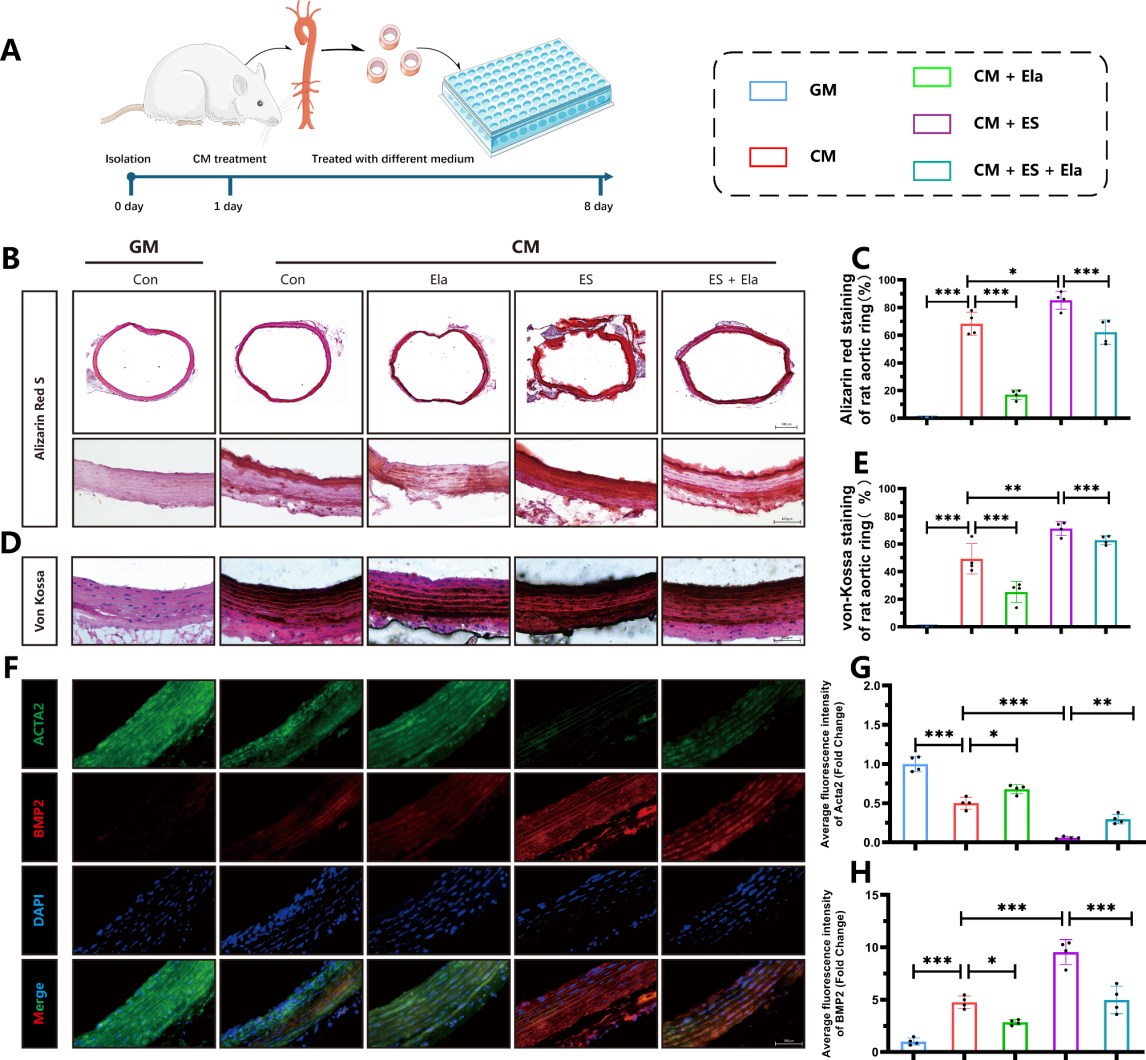
Elabela alleviates calcification of rat arterial rings **(A)** Schematic graph of culturing aortic rings. **(B-E)** Representative images and quantitative analysis of Alizarin red (**B**, **C**) (Scale bar = 500µm) and von-Kossa staining (**D**, **E**) (Scale bar = 100µm) in sections of rat aortic rings (n = 4 per group). **(F-H)** Representative fluorescent immunohistochemistry staining images and quantitative analysis of Acta2 (Green), BMP2 (Red) and DAPI (Blue) in rat aortic rings. Scale bar = 100µm, n = 4 per group. Con, Control; GM, growth medium; CM, calcifying medium; Ela, Elabela; ES, elesclomol; BMP2, bone morphogenetic protein-2; Acta2, alpha-smooth muscle Actin; DAPI, 4’,6-diamidino-2-phenylindole. *P < 0.05, **P < 0.01, ***P < 0.001

To further confirm the protective effects of Elabela against cuproptosis and vascular calcification, both in vivo model of VitD3-overloaded mice (Fig. 3) and ex vivo model of rat aortic rings were established (Fig. 4). Interestingly, von-Kossa staining (Fig. 3A), calcium content assays (Fig. 3C) and ALP activity analysis (Fig. 3D) illustrated that treatment with cuproptosis activator elesclomol aggravated calcification in aortas of VitD3-treated mice, which was remarkably blocked by Elabela administration. In addition, Elabela intervention reversed elesclomol-suppressed Acta2 and SM22α, and inhibited the elevated BMP2, Runx2, and Slc31a protein levels in mice aortas, as evidenced by Western Blotting analysis (Fig. 3E) and immunofluorescence staining (Fig. 3F), respectively. Next, rat aortic rings were incubated with GM, CM or CM in the presence of Elabela or elesclomol (Fig. 4). As expected, the ex vivo data indicated that Elabela mitigated calcification exacerbated by elesclomol by decreasing calcium deposits (Fig. 4B-E) downregulating BMP2 and upregulating Acta2 (Fig. 4F-H) in rat arterial rings. Therefore, these findings imply the inhibitory role of Elabela in cuproptosis-associated exacerbation of vascular calcification, emphasizing its potential as an effective therapeutic target in combating vascular calcification.

### Elabela is a negative regulator for cuproptosis and calcification in high phosphate induced VSMCs

**Fig. 5:**
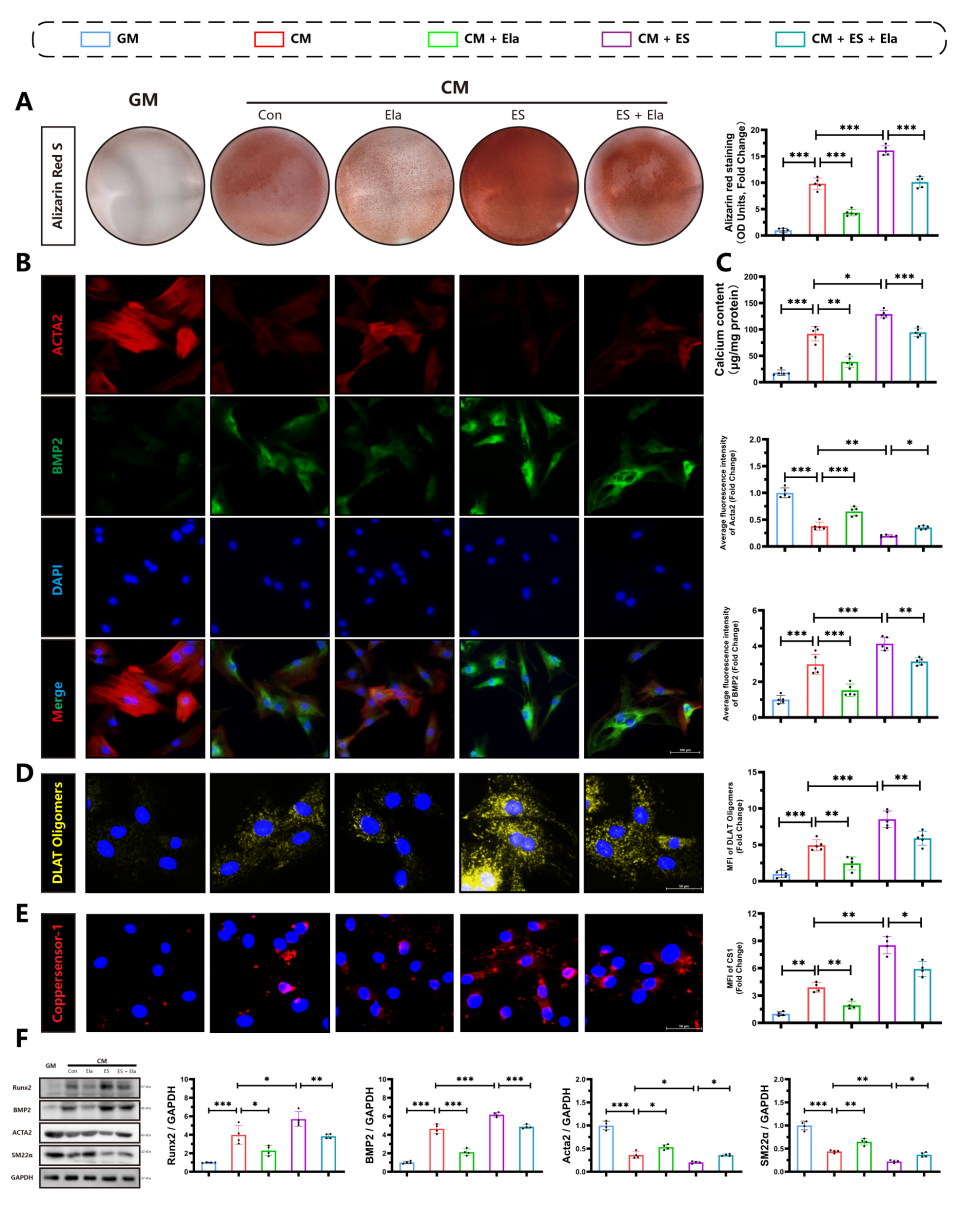
Targeting Elabela attenuates osteogenic differentiation of VSMCs **(A)** Calcium deposition in VSMCs was stained with Alizarin red solution, n = 5 per group. **(B)** Representative immunofluorescence staining images of Acta2 (Red), BMP2 (Green) and DAPI (Blue). Scale bar = 100µm, n = 5 per group. **(C)** Quantification of calcium deposition in VSMCs treated with GM or Elabela, ES with or without CM stimulation for 7 days, n = 5 per group. **(D)** Representative images of DLAT oligomer immunofluorescence imaging and quantification, Scale bar = 50µm, n = 5 per group. **(E)** Representative staining images and quantifications of CS-1, Scale bar = 50µm, n = 4 per group. **(F)** Representative western blot images and quantifications of BMP2, Acta2, Runx2 and SM22α in VSMCs, n = 4 per group. Control; GM, growth medium; CM, calcifying medium; Ela, Elabela; ES, elesclomol; Runx2, runt-related transcription factor 2; BMP2, bone morphogenetic protein-2; CS-1, Coppersensor-1; Acta2, alpha-smooth muscle actin; SM22α, smooth muscle 22 alpha; DAPI, 4’,6-diamidino-2-phenylindole. *P < 0.05, **P < 0.01, ***P < 0.001

To confirm these findings in vitro, primary rat VSMCs were isolated using previously established protocols(Chen et al. 2023a, b, c, d). The cells were characterized by using immunofluorescence staining for Acta2 (Fig S5). We next treated rat VSMCs, with Elabela or elesclomol, to investigate the effect of Elabela on cuproptosis and calcification in vitro. Alizarin red staining (Fig. 5A) and calcium content assays (Fig. 5C) revealed that osteogenic differentiation of VSMCs was induced by CM, and Elabela significantly reduced calcium deposition in VSMCs. Corresponding with the results obtained in vivo, immunofluorescence staining (Fig. 5B) and Western blot analysis (Fig. 5F) demonstrated that Elabela treatment interrupted CM-induced upregulation of osteogenic markers including Runx2 and BMP2 and downregulation of contractile factors including Acta2 and SM22α expressions in cultured primary rat VSMCs. Interestingly, stimulation with CM exacerbated the aggregation of DLAT (Fig. 5D) and increased the levels of copper ion (Fig. 5E) in VSMCs, and treatment with Elabela or TTM (Fig S7), a copper chelator, attenuated the aggregation of DLAT and copper. Remarkably, vascular calcification and cuproptosis were worsened following exposure to elesclomol, and Elabela effectively counteracted the pro-osteogenic effects of cuproptosis activation. Overall, these findings suggest that Elabela dampens the osteogenic differentiation of VSMCs associated with activation of cuproptosis.

### Elabela alleviates mitochondrial injury and senescence in VSMCs

**Fig. 6:**
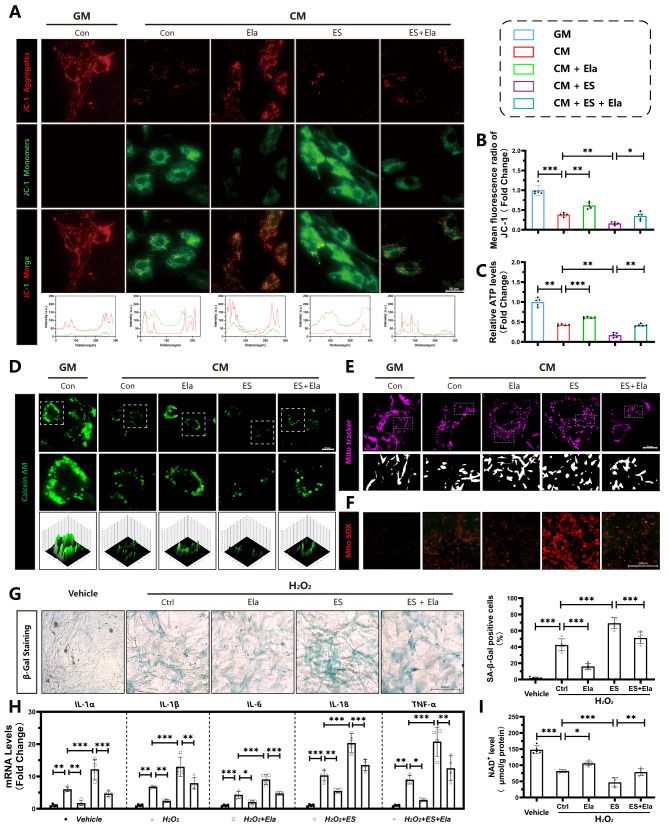
Elabela mitigates mitochondrial injury and senescence in VSMCs **(A**,**B)** Fluorescence microscopy images of JC-1 staining in VSMCs. The ratio of JC-1 Aggregates (Red) to JC-1 Monomers (Green) was calculated. Scale bar = 50 μm, *n* = 5 per group. **(C)** ATP concentration determination of VSMCs, *n* = 5 per group. **(D)** Representative images of Calcein AM staining (Green). Scale bar = 50 μm. **(E)** Representative images of MitoTracker (Magenta). Scale bar = 25 μm. **(F)** Representative images of MitoSOX (Red). Scale bar = 200 μm. **(G)** Representative β-galactosidase (β-gal) staining images and quantitative analysis of the positive cells. Scale bar = 200 μm, *n* = 5 per group. **(H)** qRT-PCR analysis of senescence-associated secretory phenotype markers expression (IL-1α, IL-1β, IL-6, IL-18, TNF-α,) in VSMCs with different treatment, *n* = 4 per group. **(I)** NAD^+^ levels in VSMCs, *n* = 5 per groups, Control; GM, growth medium; CM, calcifying medium; Ela, Elabela; ES, elesclomol; ALP, alkaline phosphatase; L-1α, interleukin 1 alpha; IL-1β, interleukin 1 beta; IL-6, interleukin 6; IL-18, interleukin 18; TNF-α, tumor necrosis factor alpha; NAD^+^, nicotinamide adenine dinucleotide; ATP, adenosine triphosphate; H_2_O_2_, hydrogen peroxide. *P < 0.05, **P < 0.01, ***P < 0.001

Given that mitochondrial injury and senescence are related to the progression of vascular calcification(Li et al. 2024; Zhu et al. 2023), we then investigated whether Elabela inhibits vascular calcification by targeting mitochondrial injury and senescence. To assess mitochondrial function, we measured the mitochondrial membrane potential using JC-1, Calcein AM staining, ATP levels, MitoTracker and MitoSOX staining. The fluorescence intensity ratio of Red to Green was higher in the group treated with Elabela in the presence of CM compared to the CM group, indicating improved mitochondrial function (Fig. 6A, B). Mitochondrial permeability transition pore over-opening, a marker of mitochondrial injury, was also reduced by Elabela treatment (Fig. 6D). Meanwhile, incubation with CM resulted in the promotion of mitochondrial fission in VSMCs, as indicated by a higher ratio of fragmented mitochondria and a reduction in mitochondrial length (Fig. 6E). Correspondingly, following Elabela treatment, mitochondrial fragmentation was inhibited, and mitochondrial length was restored in VSMCs (Fig. 6E). Interestingly, Elabela was able to protect VSMCs from increased MitoSOX generation (Fig. 6F), reduced ATP production (Fig. 6C), and upregulated mitochondrial fragmentation caused by elesclomol (Fig. 6E). Senescent VSMCs are known to contribute to arterial calcification(Li et al. 2024). In addition, Elabela expression was decreased in vascular calcification associated with aging, as we demonstrated (Fig. 1). Thus, we further investigated the role of Elabela in VSMC senescence. Cellular senescence was induced by H_2_O_2_ treatment, which exacerbated CM induced VSMC calcification (Fig S8). Elabela treatment significantly reduced the percentage of senescent cells, as evidenced by a decrease in β-galactosidase-positive cells (Fig. 6G). Moreover, Elabela inhibited mRNA levels of pro-inflammatory cytokines involved in senescent-related secretion profiles in VSMCs, including IL-1α, IL-1β, IL-6, IL-18 and TNF-α (Fig. 6H), as well as prevented the calcifying mediated decrease in NAD^+^ levels (Fig. 6I). More importanly, treatment with the elesclomol resulted in an increase in the percentage of senescent cells, exacerbation of senescent-related secretion profiles, and decrease in NAD^+^ levels. Remarkably, these effects could be counteracted by Elabela treatment. Overall, our findings suggest that Elabela has a protective effect against mitochondrial injury and senescence in VSMCs under calcification conditions.

### Inhibition of PPAR-γ abolishes the protective effect of Elabela in vascular calcification and cuproptosis

**Fig. 7:**
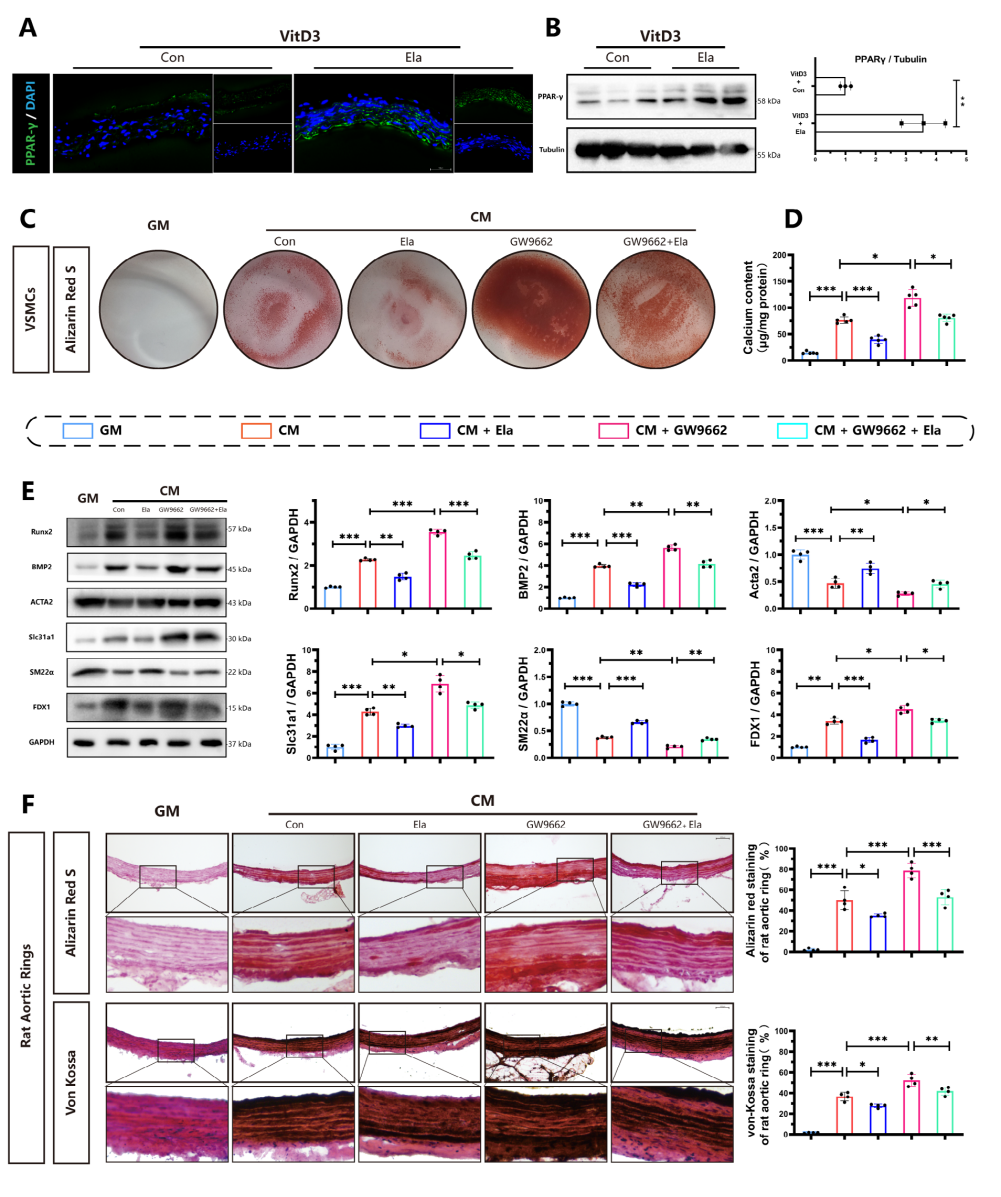
Inhibition PPAR-γ blunts the protective effects of Elabela on vascular calcification and cuproptosis **(A)**Representative immunofluorescence staining and quantitative analysis of PPAR-γ in the aortic sections. **(B)**Western blots for PPAR-γ protein levels in aortas from VitD3 and VitD3 + Elabela treated mice (n = 3). **(C)** VSMCs were treated with GM, CM, Elabela or together with PPAR-γ inhibitor, GW9662, for 7 days. Then VSMCs were stained by Alizarin red S. **(D)** Quantification of calcium deposition in VSMCs (n = 5). **(E)** Representative western blots images and quantification of Slc31a1, FDX1, BMP2, Acta2, Runx2 and SM22α (n = 4). **(F)** Representative images of Alizarin red S and von-Kossa staining in sections of aortic rings (Scale bar = 100µm, n = 4). Con, Control; VitD3, Vitamin D3; GM, growth medium; CM, calcifying medium; Ela, Elabela; PPAR-γ, peroxisome proliferator activated receptor gamma; Runx2, runt-related transcription factor 2; BMP2, bone morphogenetic protein-2; Acta2, alpha-smooth muscle actin; SM22α, smooth muscle 22 alpha; FDX1, ferredoxin 1; Slc31a1, solute carrier family 31 member 1; DAPI, 4’,6-diamidino-2-phenylindole. *P < 0.05, **P < 0.01, ***P < 0.001

PPAR-γ plays an important role in vascular calcification, as evidenced by both our experimental results (Fig. 2) and previous studies (Kukida et al. 2019). In addition, apelin, which shares the same receptor of Elabela, has been identified as a target that modulates the transcriptional activity of PPAR-γ(Alastalo et al. 2011). Therefore, we decided to investigate whether Elabela inhibits vascular calcification and cuproptosis via PPAR-γ. Increased levels of PPAR-γ protein were observed in aortic tissues of Elabela-treated mice in the presence of VitD-3, as demonstrated by immunofluorescence (Fig. 7A) and Western blot analyses (Fig. 7B). Interestingly, when PPAR-γ was inhibited using GW9662, there was a significant aggravation of calcification in primary rat VSMCs (Fig. 7C). Additionally, the protective effects of Elabela in the mitigation of calcification and cuproptosis were largely abolished in the presence of GW9662, as evidenced by changes in the protein levels of Acta2, SM22α, Runx2, BMP2, FDX1, and Slc31a1 (Fig. 7E), as well as results from Alizarin red staining (Fig. 7C) and calcium content assays (Fig. 7D). Consistent with these findings, GW9662 treatment significantly aggravated calcification in the presence of Elabela, as shown by Alizarin red and von Kossa staining in rat aortic rings (Fig. 7F). Taken together, these results strongly support the conclusion that Elabela alleviates vascular calcification through the PPAR-γ signaling.

### Elabela inhibits vascular calcification via the modulation of ATP7a

**Fig. 8:**
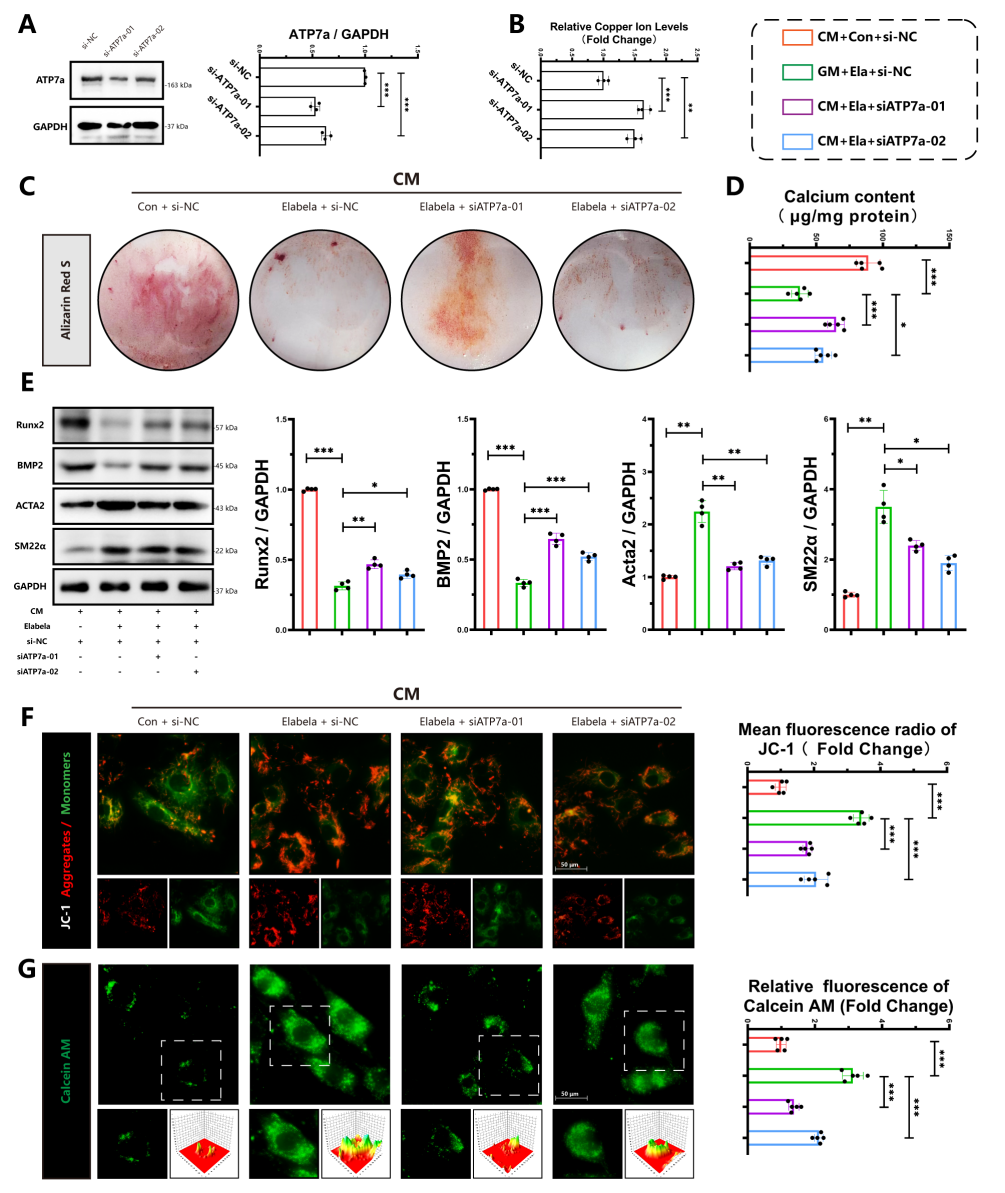
Elabela inhibits vascular calcification in VSMCs via modulation ATP7a **(A)** Representative western blots images and quantification for ATP7a protein expression (*n* = 3). **(B)** Measurement of copper content in VSMCs, *n* = 3. **(C)** Representative Alizarin red staining images of VSMCs. **(D)** Quantification of calcium deposition in VSMCs (*n* = 5). **(E)** Representative western blots and quantification of BMP2, Acta2, Runx2 and SM22α in VSMCs (*n* = 4). **(F)** Representative Fluorescence microscopy images of JC-1 staining in VSMCs (Scale bar = 50 μm, *n* = 5). The ratio of JC-1 Aggregates (Red) to JC-1 Monomers (Green) was calculated. **(G)** Representative images and quantitative analysis of Calcein AM staining in VSMCs (Green) (Scale bar = 50 μm, *n* = 5). CM, calcifying medium; Ela, Elabela; ES, elesclomol; Runx2, runt-related transcription factor 2; BMP2, bone morphogenetic protein-2; Acta2, alpha-smooth muscle actin; SM22α, smooth muscle 22 alpha; siRNA, small interfering RNA; ATP7a, ATPase copper transporting alpha. *P < 0.05, **P < 0.01, ***P < 0.001

Copper homeostasis plays a crucial role in the development of vascular dysfunction(Zhong et al. 2024). Elabela was found to prevent the worsening of vascular calcification caused by elesclomol. We next investigated whether decreased cellular copper efflux blocks the protective effect of Elabela. Si-RNA was used to transfect VSMCs targeting ATP7a, a key regulator of intracellular copper levels(Kaler 2011), to reduce copper efflux, and transfection efficiency was verified by WB analysis (Fig. 8A). Compared with si-NC group, the copper content in VSMCs was increased following ATP7a inhibition (Fig. 8B). Results from alizarin red staining (Fig. 8C), calcium content assay (Fig. 8D), and protein levels of Acta2, SM22α, Runx2, and BMP2 (Fig. 8E) indicated that Elabela supplementation could effectively reduce calcification and osteogenic differentiation induced by CM in VSMCs. Conversely, the knockdown of ATP7a using siRNA diminished the protective effects of Elabela. Furthermore, JC-1 (Fig. 8F) and Calcein AM (Fig. 8G) staining revealed that the protective effects of Elabela on mitochondria were also blocked by ATP7a knockdown, revealing that Elabela exerts its anti-cuproptosis and anti-calcification properties through ATP7a via the regulation of the efflux of intracellular copper.

## Discussion

Copper is an important mineral that plays a critical role in various physiological processes within the human body(Chen et al. 2022a, b; Tsang et al. 2021). Maintaining a balance of copper homeostasis is essential for cellular functions such as energy production and antioxidant defense (Chen et al. 2022a, b, 2023a, b, c, d). Cuproptosis, a type of cell death triggered by disrupted copper levels, can result in mitochondrial dysfunction, over-production of reactive oxygen species, and ultimately cell death(Tsvetkov et al. 2022). In the present study, we uncover a novel finding that Elabela inhibits the osteogenic differentiation of VSMCs and mitigates vascular calcification by suppressing cuproptosis. An accumulation of FDX1, a key mediator of cuproptosis(Tsvetkov et al. 2022), and higher copper ion levels were observed in calcified aortas compared to uncalcified aortas. Notably, qRt-PCR analysis demonstrated a clear inverse relationship between Elabela levels and the extent of vascular calcification, indicating a potential role of Elabela in modulating the calcification process. Additionally, intervention with Elabela resulted in decreased calcification levels, mitochondrial abnormalities, and age-related characteristics. Further in vitro studies confirmed that Elabela treatment downregulated the expression of FDX1 and SLC31a1 in VSMCs under CM conditions. Interestingly, induction of cuproptosis by elesclomol promoted calcification in VSMCs, as well as in rat aortic rings and mouse model, which were alleviated by Elabela treatment. Mechanistically, the inhibitory effects of calcification and cuproptosis by Elabela were partially associated with PPAR-γ and could be blocked by the PPAR-γ inhibitor, GW9662, indicating that Elabela exerts its vascular protective effects partly through the activation of PPAR-γ signaling. Surprisingly, blocking intracellular copper efflux by ATP7a-inhibition partially reversed the inhibitory effects of Elabela on VSMC osteogenic differentiation, suggesting that Elabela exerts its beneficial roles in calcification through cuproptosis signaling pathway. Collectively, these findings underscore the significance of Elabela in the regulation of vascular calcification and propose that Elabela could hold promise in preventing or treating vascular calcification and cuproptosis in future.

The protein Elabela, also known as APELA, has been implicated to play crucial roles in a variety of biological processes(Chapman et al. 2023; Monastero et al. 2023). Elabela is pivotal in regulating cardiovascular development, functioning, and maintaining cardiovascular homeostasis. Together with apelin and APJ, Elabela constitutes the apelin-APJ system, which serves as a comprehensive regulator of cardiovascular physiology(Wang et al. 2024). Both clinical and experimental studies have underscored the various cardioprotective properties of the Elabela-apelin-APJ axis(Monastero et al. 2023). A recent research has unveiled Elabela’s ability to impede the phenotypic transition of VSMCs in spontaneously hypertensive rats(Ye et al. 2022), and Apelin-13 has been shown to mitigate high glucose-induced calcification of VSMCs(Zhang et al. 2020). Nonetheless, the detailed mechanisms of the role of Elabela in aging associated vascular calcification remain insufficiently elucidated. Of interest, we observed that Elabela mRNA levels were significantly diminished in VSMCs treated with CM and in arteries with age-related calcification. Elabela notably reduced vascular calcification in VitD3-induced mouse and in ex vivo vascular tissue culture. Furthermore, Elabela exhibited a promising ability to impede the transition of VSMCs into osteoblast-like cells, which is a crucial aspect in the pathogenesis of vascular calcification. These findings suggest that Elabela may offer protective effects against VSMC transformation and vascular calcification development, marking the first evidence directly linking Elabela to the repression of vascular calcification.

Moreover, Elabela has been identified as having significant protective effects against aging-related diseases such as cardiovascular diseases(Ye et al. 2022; Yang et al. 2017; Zhang et al. 2022), neurodegenerative disorders(Xu et al. 2023), and metabolic syndromes(Hong et al. 2023). Research studies have shown that Elabela can prevent neurons from degeneration, decrease brain inflammation, and enhance cognitive function(Xu et al. 2023; Zhang et al. 2023a, b). It has also been noted that serum levels of Elabela decline in individuals with hypertension and suggested its potential as a marker for hypertension-related kidney impairments(Tian et al. 2024). To investigate the role of Elabela in aging, H_2_O_2_ was utilized to induce cellular senescence in VSMCs. Results revealed a lower percentage of β-gal positive cells in Elabela-treated group compared to the control group in the presence of CM. Additionally, Elabela treatment reduced the expression of senescence-associated secretory phenotype (SASP) factors that are known to promote inflammation and tissue dysfunction during cellular senescence. Furthermore, Elabela treatment increased levels of NAD^+^, a vital molecule involved in cellular energy metabolism and aging regulation, suggesting a protective effect of Elabela against aging-induced damage in VSMCs. Overall, our findings indicate that Elabela has a protective effect against aging-induced damage in VSMCs.

Mitochondria are crucial organelles responsible for producing ATP through the TCA cycle and oxidative phosphorylation(Martínez-Reyes et al. 2016; Martínez-Reyes and Chandel 2020). Mitochondrial dysfunction induces impaired energy metabolism and oxidative stress, and promotes vascular calcification by affecting the activity of calcification regulatory proteins, promoting inflammation and cellular senescence(Pescatore et al. 2019; Lee et al. 2020a, b). Our results indicated that Elabela treatment effectively protected mitochondria from damage caused by high phosphate levels, evidenced by improved mitochondrial membrane potential, reduced opening of the MPTP, and restored ATP levels. The protective role of Elabela in mitochondrial impairments and its potential as a therapeutic agent for anti-vascular calcification highlights the need for further research to explore its clinical applications.

Cuproptosis is a specific form of cell death that is activated by excessive high levels of copper in various cardiovascular and liver diseases (Tsvetkov et al. 2022). A key feature of cuproptosis is its ability to generate oxidative stress and disrupt mitochondrial function (Chen et al. 2022a, b, 2023a, b, c, d, 2024; Tsvetkov et al. 2022). Since its discovery in 2022(Tsvetkov et al. 2022), mounting evidence has indicated that cuproptosis plays a significant role in vascular diseases. Additionally, recent research suggested that excessive copper uptake can trigger oxidative stress and inflammation, leading to endothelial dysfunction in individuals with diabetes(Zhong et al. 2024). But the mechanisms by which cuproptosis affect vascular calcification and the associated regulatory factors remain unclear. In the present investigation, we first determined if cuproptosis is involved in vascular calcification. Notably, our findings revealed a significant increase in FDX1 under calcification conditions in the presence of aggregation of copper, with mitochondrial injury, cell aging and decreased Elabela RNA levels. Fascinatingly, the activation of cuproptosis by elesclomol, a copper ionophore, significantly increased the calcification area of arteries in VitD3-induced rats and mice, as well as rat arterial rings. Furthermore, in vivo experiments confirmed that elesclomol worsened CM-induced calcium deposition, mitochondrial damage, and cellular senescence. Noteworthy, following exogenous supplementation of Elabela, there was a significant reduction in the protein level of FDX1. Particularly interesting, Elabela was found to counteract the effects of elesclomol on calcium deposition, mitochondrial damage, and VSMC aging. It has been demonstrated that ATP7a plays a vital role in the transportation of copper out of the cell to maintain copper homeostasis(Kaler 2011). When ATP7a was knocked down by using siRNA, the efflux mechanism was blocked, thereby contributing to a notable decrease in the protective effects of Elabela on mitochondria and vascular calcification. To the best of our knowledge, this work is the first to provide direct evidence that the Elabela-Apelin-APJ system attenuates cuproptosis and vascular calcification during cardiovascular damage and aging.

Peroxisome proliferator-activated receptor gamma (PPAR-γ) is a nuclear receptor that plays a pivotal role in regulating lipid metabolism. Its involvement in cardiovascular diseases has garnered significant attention in recent years. PPAR-γ activation has been exhibited to exert protective effects on the cardiovascular system by enhancing insulin sensitivity, reducing inflammation, and promoting the differentiation of adipocytes. The depletion or insufficiency of PPAR-γ has been linked to the acceleration of various vascular conditions such as atherosclerosis, aortic aneurysm, and dissection(Kukida et al. 2019; Marchesi et al. 2013; Halabi et al. 2008). Interestingly, the dysregulation of PPAR-γ signal transduction was identified as the central pathological mechanism in vascular calcification(Kukida et al. 2019). Activation of PPAR-γ has been implicated to impede the transformation of VSMCs into osteoblast-like cells, which plays a key role in the formation of calcium and phosphate deposits in the aorta walls(Woldt et al. 2012). Recently, PPAR-γ activator has been revealed to exert benefits through the suppression of programmed cell death, particularly in early stages of diseases (Montaigne et al. 2021). However, the effect of Elabela on PPAR-γ remains unexplored. Our findings reveal an increase in PPAR-γ expression in aorta tissue after Elabela treatment in the presence of VitD-3. Furthermore, when a PPAR-γ inhibitor was administered, the protective function of Elabela against vascular calcification and its capability to prevent cuproptosis were hindered. Taken together, PPAR-γ activation may be crucial for the beneficial effects of Elabela in cuproptosis and vascular calcification.

## Conclusion

**Fig. 9:**
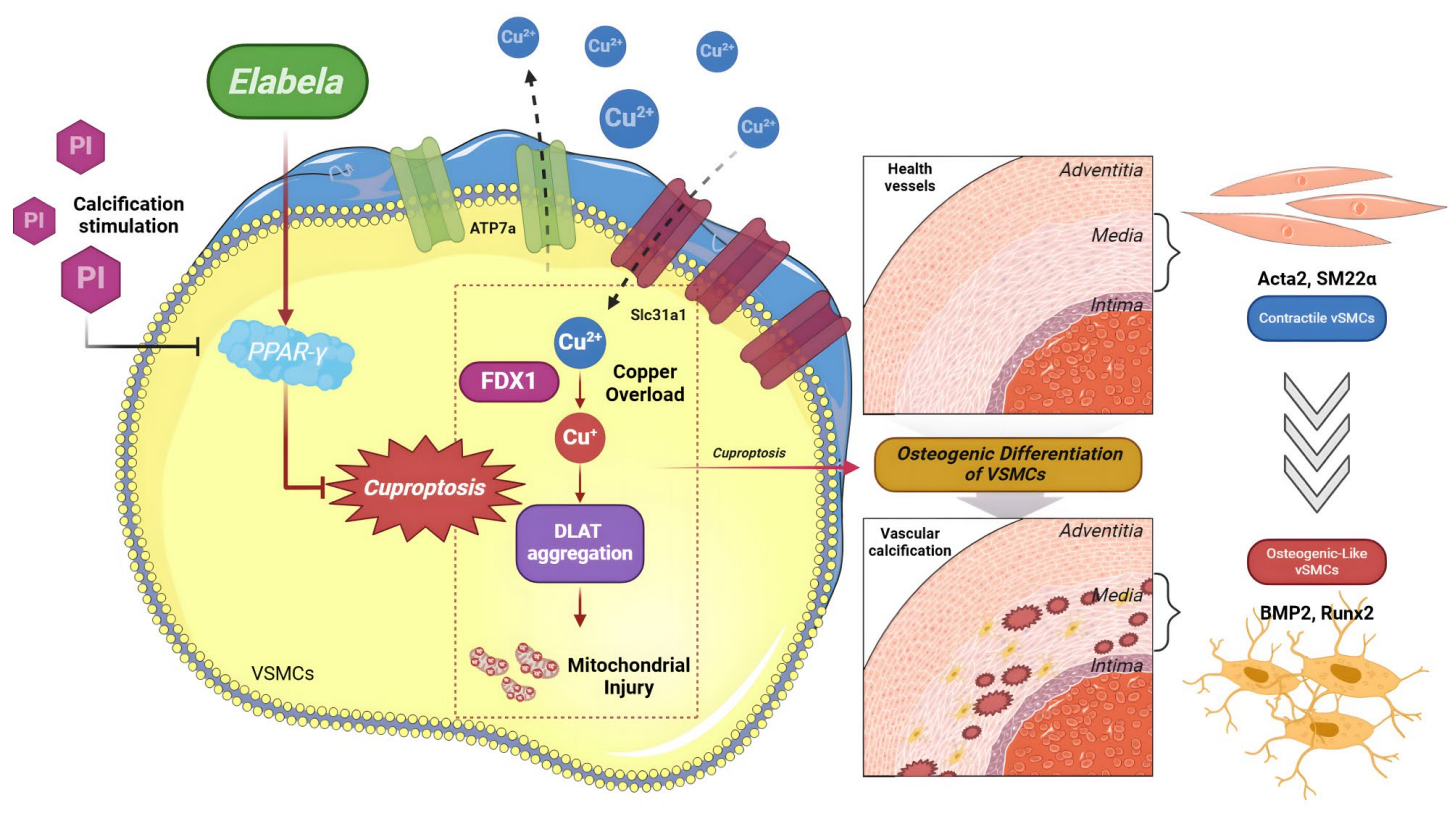
Schematic diagram illustrating the molecular mechanisms underlying the protective effect of Elabela in vascular calcification Upregulation of FDX1 and Slc31a1 during vascular calcification pathology leads to cuproptosis in VSMCs, thereby triggering mitochondrial damage and accelerating the process of osteogenic transition of VSMCs. More importantly, Elabela alleviates cuproptosis and vascular calcification, as well as the subsequent mitochondrial damage and cellular senescence by facilitating the PPAR-γ/ATP7a signaling pathway. FDX1, ferredoxin 1; Slc31a1, solute carrier family 31 member 1; Runx2, runt-related transcription factor 2; BMP2, bone morphogenetic protein-2; Acta2, alpha-smooth muscle actin; SM22α, smooth muscle 22 alpha; Slc31a1, solute carrier family 31 member 1; ATP7a, ATPase copper transporting alpha; PPAR-γ, peroxisome proliferator activated receptor gamma

In summary, these discoveries outlined in the current work shed light on the pivotal impact of Elabela on cuproptosis and vascular calcification in VitD3-overloaded mice. Elabela has a capacity to impede cuproptosis and vascular calcification, mitigate mitochondrial injury and cellular senescence by inhibiting Runx2, BMP2 and FDX1 signaling, and activating PPAR-γ signaling (Fig. 9). Ultimately, these findings emphasize the significance of Elabela as a promising therapeutic target for tackling vascular calcification. With its diverse therapeutic benefits, the activation of Elabela-PPARγ signaling and inhibition of cuproptosis emerge as compelling subjects for further investigation and clinical exploration, offering a beacon of hope for enhanced treatment options for disorders linked to vascular calcification.

## Electronic supplementary material

Below is the link to the electronic supplementary material.

Supplementary Material 1

## Data Availability

No datasets were generated or analysed during the current study.
