# Supplementary material for: Elabela alleviates cuproptosis and vascular calcification in vitaminD3- overloaded mice via regulation of the PPAR-γ /FDX1 signaling
Source: Mol Med. 2024 Nov 20;30:223. doi: 10.1186/s10020-024-00997-3 (PMC11577739; doi:10.1186/s10020-024-00997-3)
Supplement: Supplementary file 1 — Supplementary Material 1 [file 10020_2024_997_MOESM1_ESM.docx]

**Elabela Alleviates Cuproptosis and Vascular Calcification in VitaminD3- Overloaded Mice via Regulation of the PPAR-γ /FDX1 Signaling**

**Rui-Qiang Qi ^a,b,†^, Yu-Fei Chen ^a,b,†^, Jing Cheng^b,†^, Jia-Wei Song^a,b^, Yi-Hang Chen^a^, Si-Yuan Wang^a^, Ying Liu^a,b^, Kai-Xin Yan^a^, Xiao-Yan Liu^a^, Jing Li^a^, Jiu-Chang Zhong^a,b,*^**

**^a^** Heart Center and Beijing Key Laboratory of Hypertension, Beijing Chaoyang Hospital, Capital Medical University. Beijing 100020, China.

**^b^** Department of Cardiology, Beijing Chaoyang Hospital, Capital Medical University, Beijing 100020, China.

**^†^**Authors contributed equally

**Running Head:** Elabela mitigates vascular calcification.

***Corresponding Author:** Jiu-Chang Zhong. M.D. & Ph.D.; Heart Center and Beijing Key Laboratory of Hypertension, Beijing Chaoyang Hospital, Capital Medical University, Beijing 100020; **Telephone：**+86-10-89138377; **Fax：**+86-10-85231937; **Email：**[jczhong@sina.com](mailto:jczhong@sina.com); **ORCID:** Jiu-Chang Zhong: <https://orcid.org/0000-0002-2315-3515>

**Supplementary Tables and Figures**

**Supplemental Methods**

***Bioinformatics analysis***

Microarray data from the GEO database (GSE146638) were used to identify aberrantly expressed gene of cuproptosis in rat vascular calcification induced by VitD3. Heatmap was plotted by https://www.bioinformatics.com.cn, an online platform for data analysis and visualization(1). Gene Ontology (GO) and Kyoto Encyclopedia of Genes and Genomes (KEGG) enrichment analyses were further conducted for functional analysis by Metascape(2).

***Quantification of SMCs alizarin red staining***

As previously reported in the literature(3), the plates of alizarin red staining were eluted with 10% formic acid, the absorbance was measured at 405 nm using a microplate reader.

**References**

1. Tang D*, et al.* (2023) SRplot: A free online platform for data visualization and graphing. *PLoS One* **18:** e0294236.

2. Zhou Y*, et al.* (2019) Metascape provides a biologist-oriented resource for the analysis of systems-level datasets. *Nat Commun* **10:** 1523.

3. Wang S*, et al.* (2022) Deletion of SIRT6 in vascular smooth muscle cells facilitates vascular calcification via suppression of DNA damage repair. *J Mol Cell Cardiol* **173:** 154-168.

**Supplementary Table**

**Supplementary Table S1. Sequences of primers (5'->3') used for qRT-PCR.**

| **Elabela** | forward | TCATGGGAAGGGCACTCGA |
| --- | --- | --- |
|  | reverse | ACTGCTTTCGCCGGAGATG |
| **IL-1α** | forward | AGCCTGTGTTGCTGAAGGAGATTC |
|  | reverse | CTCTGGGAAAGCTGCGGATGTG |
| **IL-1β** | forward | ATCTCACAGCAGCATCTCGACAAG |
|  | reverse | GAAGCTCCACGGGCAAGACATAG |
| **IL-6** | forward | ACTTCCAGCCAGTTGCCTTCTTG |
|  | reverse | TGGTCTGTTGTGGGTGGTATCCTC |
| **IL-18** | forward | CCTGATATCGACCGAACAGCCAAC |
|  | reverse | TCACAGATAGGGTCACAGCCAGTC |
| **TNF-α** | forward | CCAGAACTCCAGGCGGTGTC |
|  | reverse | GGCTACGGGCTTGTCACTCG |
| **FDX1** | forward | ACAGTCCACTTCAAGAACCGAGATG |
|  | reverse | ACAGGTAGAGCAAGCCAAAGTCC |
| **SLC31a** | forward | CCACACGGACGACAACATCAC |
|  | reverse | GAAGGTCATAGGCATCATCATCTCG |
| **GAPDH** | forward | AAGTTCAACGGCACAGTCAAGG |
|  | reverse | ACGCCAGTAGACTCCACGACAT |

IL-1α, interleukin 1 alpha; IL-1β, interleukin 1 beta; IL-6, interleukin 6; IL-18, interleukin 18; TNF-α, tumor necrosis factor alpha; FDX1, ferredoxin 1; Slc31a1, solute carrier family 31 member 1; GAPDH, Glyceraldehyde-3-Phosphate Dehydrogenase.

**Supplementary Table S2. Sequences of si-RNA.**

| **si-ATP7-1** | Sense | GCUCUUCUUCACAAUGCUATT |
| --- | --- | --- |
|  | Antisense | UAGCAUUGUGAAGAAGAGCTT |
| **si-ATP7-2** | Sense | GCUCAGAAUGGCAUACUUATT |
|  | Antisense | UAAGUAUGCCAUUCUGAGCTT |

ATP7a, ATPase copper transporting alpha.

**Supplementary Table S3. Primarily antibody used for Western Blots**

| **Antibody** | **Source** | **Host** |
| --- | --- | --- |
| Runx2 | Proteintech | Rabbit |
| BMP2 | Abclonal | Rabbit |
| Acta2 | Boster | Mouse |
| SM22α | Abclonal | Rabbit |
| FDX1 | Proteintech | Rabbit |
| Slc31a1 | Abclonal | Rabbit |
| PPARγ | Proteintech | Rabbit |
| DLAT | Proteintech | Rabbit |
| ATP7a | Abmart | Rabbit |
| GAPDH | Proteintech | Rabbit |
| Tubulin | Proteintech | Rabbit |

Runx2, runt-related transcription factor 2; BMP2, bone morphogenetic protein-2; Acta2, alpha-smooth muscle actin; SM22α, smooth muscle 22 alpha; FDX1, ferredoxin 1; Slc31a1, solute carrier family 31 member 1; PPARγ, peroxisome proliferator-activated receptors gamma; DLAT, Dihydrolipoamide acetyltransferase; ATP7a, ATPase Copper Transporting Alpha; GAPDH, Glyceraldehyde-3-Phosphate Dehydrogenase.

**Supplementary Figures and** **Figure Legends**

**
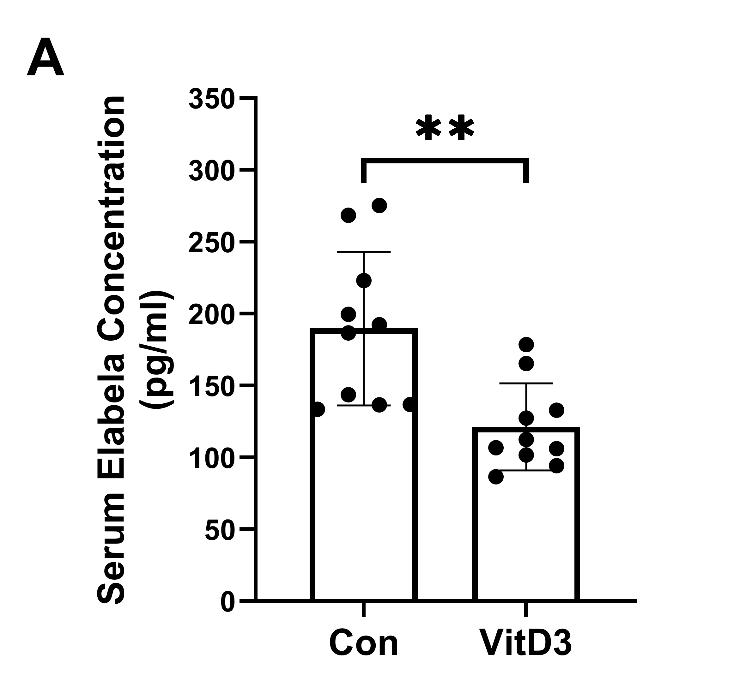
**

**Supplementary Figure S1.** **Elabela levels are decreased in serum during vitamin D3-induced vascular calcification.** C57BL/6J mice were administered a continuous injection of Vitamin D3 for 3 days to induce vascular calcification. After 14 days, the mice were euthanized, and serum samples were collected. **(A)**Serum levels of Elabela in mice were measured using an ELISA assay. n=10 per group. **P < 0.01.


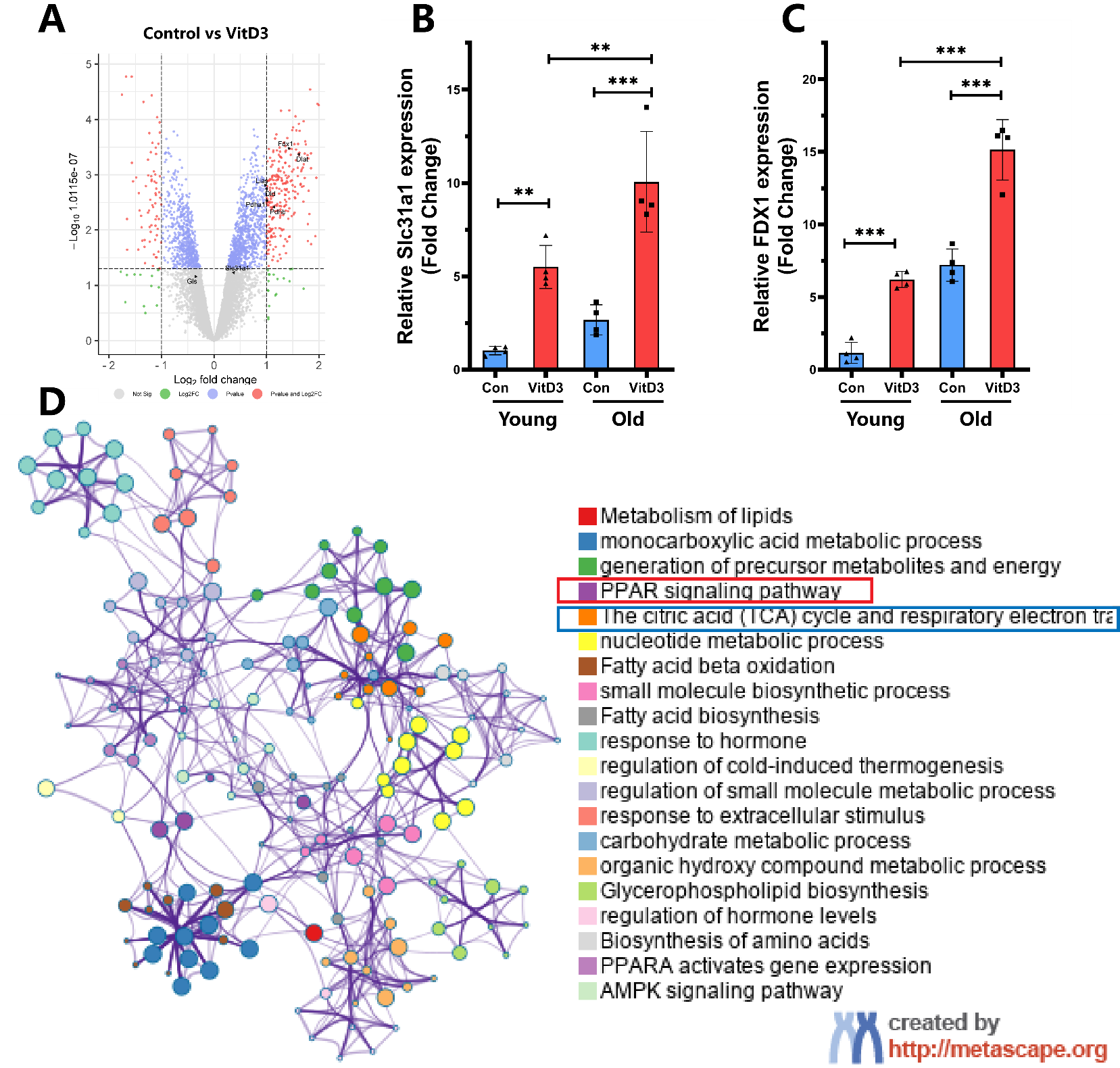


**Supplementary Figure S2. Analysis of differentially expressed genes related to cuproptosis and functional enrichment analysis in VitD3-induced vascular calcification in rats. (A)** The identified cuproptosis-related genes displayed in a volcano plot showed FDX1 and Slc31a1 were upregulated. **(B, C)** The mRNA levels of Slc31a1 (B) and FDX1 (C) in aorta of rat were examined by qRT-PCR (n=4 per group). **(D)** Networking of differential expressed genes in VitD3-induced vascular calcification (GSE146638) was visualized with Cytoscape. Con, Control; VitD3, Vitamin D3; FDX1, ferredoxin 1; Slc31a1, solute carrier family 31 member 1. ** P < 0.01, *** P < 0.001.


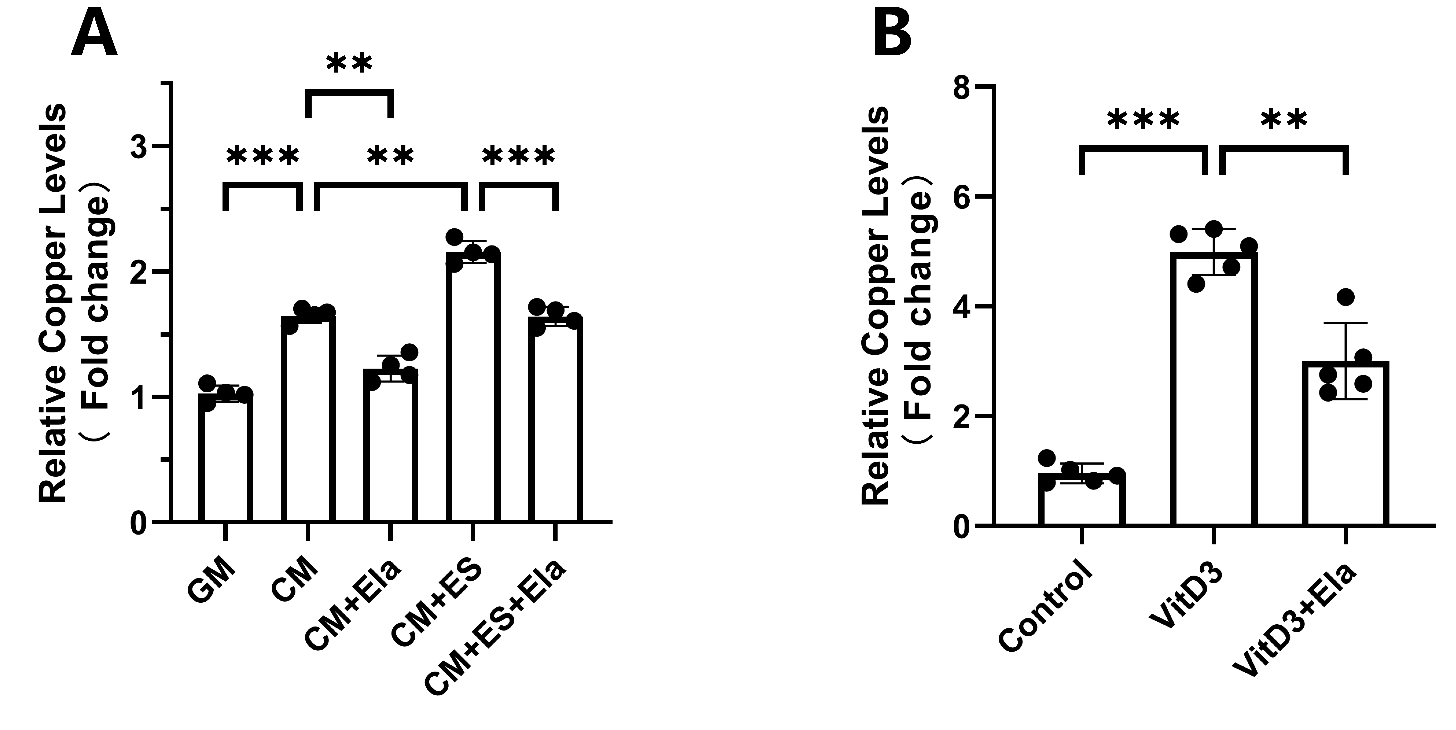


**Supplementary Figure S3. Elabela reduced copper iron overload both *in-vivo* and *in-vitro* during calcification model. (A)** Measurement of copper content in vascular tissues pretreated with Ela and ES, n=4. **(B)** Measurement of copper content in vascular tissues in VitD3-overloaded mice pretreated with and without Ela, n=5. GM, growth medium; CM, calcifying medium; VitD3, Vitamin D3; Ela, Elabela; ES, elesclomol; *P < 0.05, **P < 0.01, **P < 0.001.


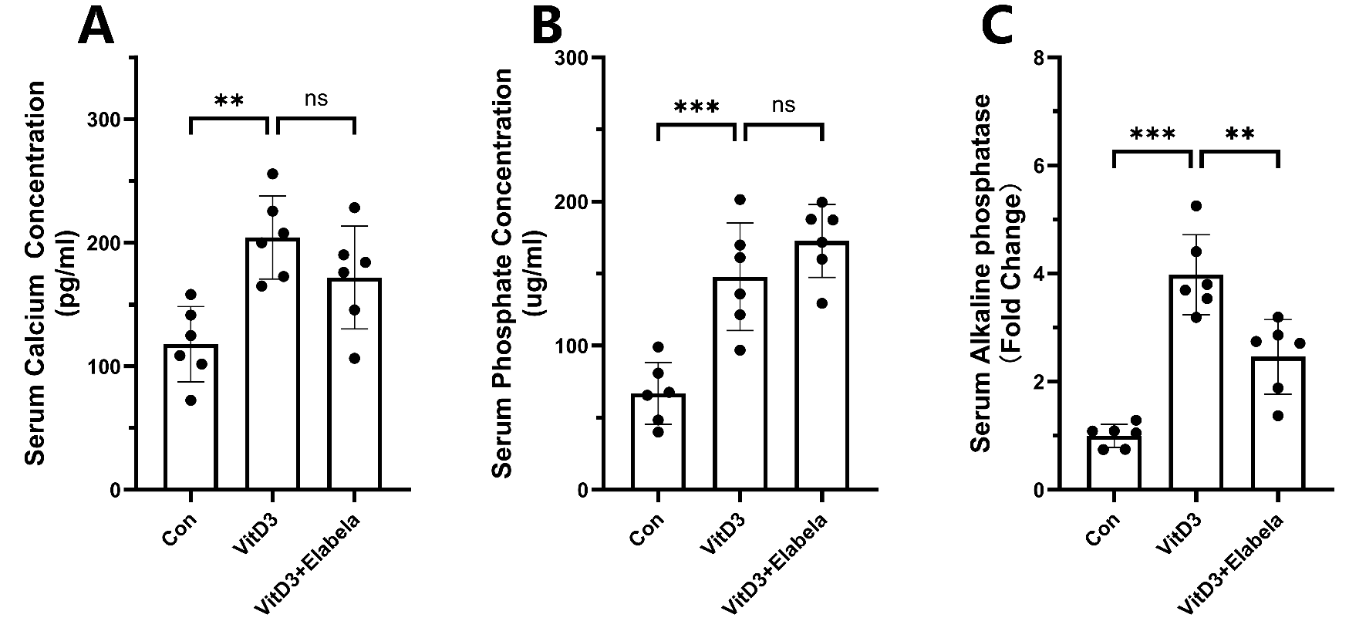


**Supplementary Figure S4. Serum levels of Calcium,** **Phosphate and ALP were measured in VitD3-overloaded mice pretreated with or without Elabela.** C57BL/6J mice were administered a continuous injection of Vitamin D3 for 3 days to induce vascular calcification. After 7 days, the mice were euthanized, and serum samples were collected. Quantification of serum calcium concentration (A), phosphate concentration (B) and Alkaline phosphatase levels (C) in VitD3-overloaded mice. Con, Control; VitD3, Vitamin D3; ns, no significance; n=6 per group. *P < 0.05, **P < 0.01, ***P < 0.001.


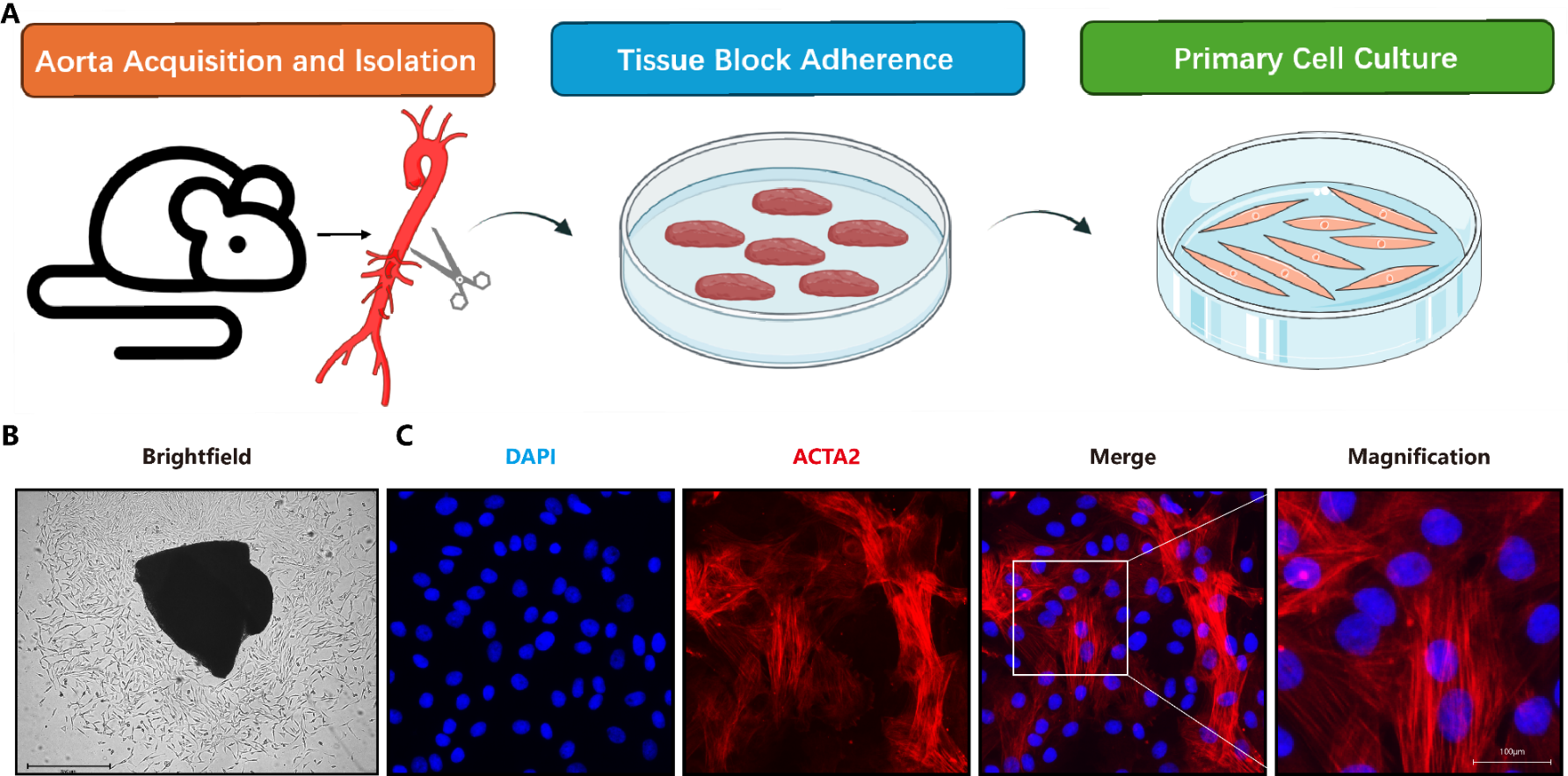


**Supplementary Figure S5. Identification of primary rat aortic vascular smooth muscle cells. (A)**Graph of rat vascular smooth muscle cell isolation. **(B)** Bright-field view of primary VSMCs. **(C)** Expression of ACTA2 was determined by a confocal microscopy. Scale bar = 100 μm. Acta2, Alpha-Smooth Muscle Actin; DAPI, 4’,6-diamidino-2-phenylindole.

**
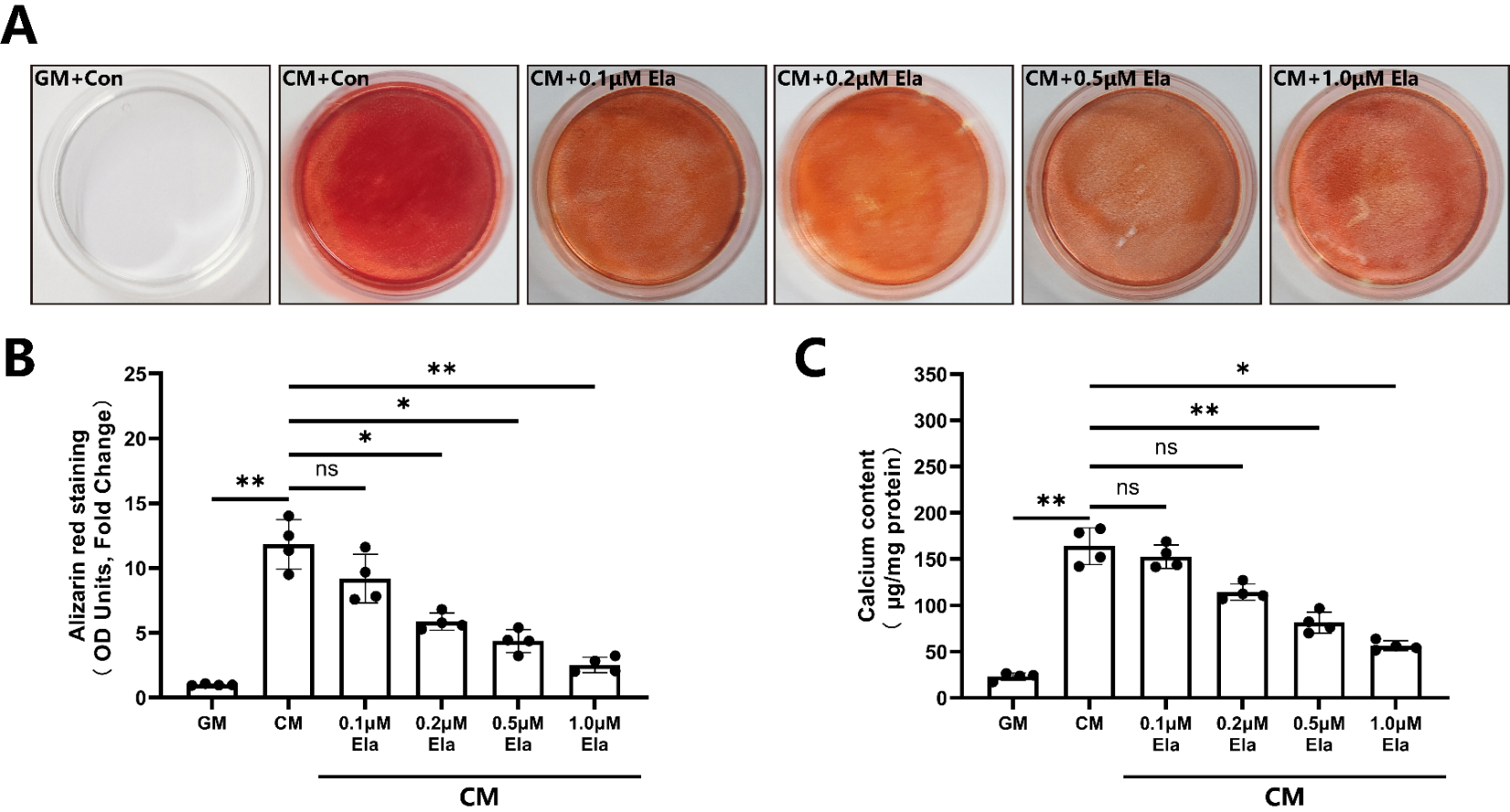
**

**Supplementary Figure S6. Elabela alleviated vascular calcification in a dose-dependent manner.** Rat VSMCs were treated with GM, CM or Elabela for 7 days. (n = 4) **(A-B)** Alizarin red staining (A) with auantitative analysis (B) was used to assess mineral deposition in primary rat VSMCs. (C) Calcium content was measured by calcium content assay. GM, growth medium; CM, calcifying medium; ns, no significance; *P < 0.05; **P < 0.01.


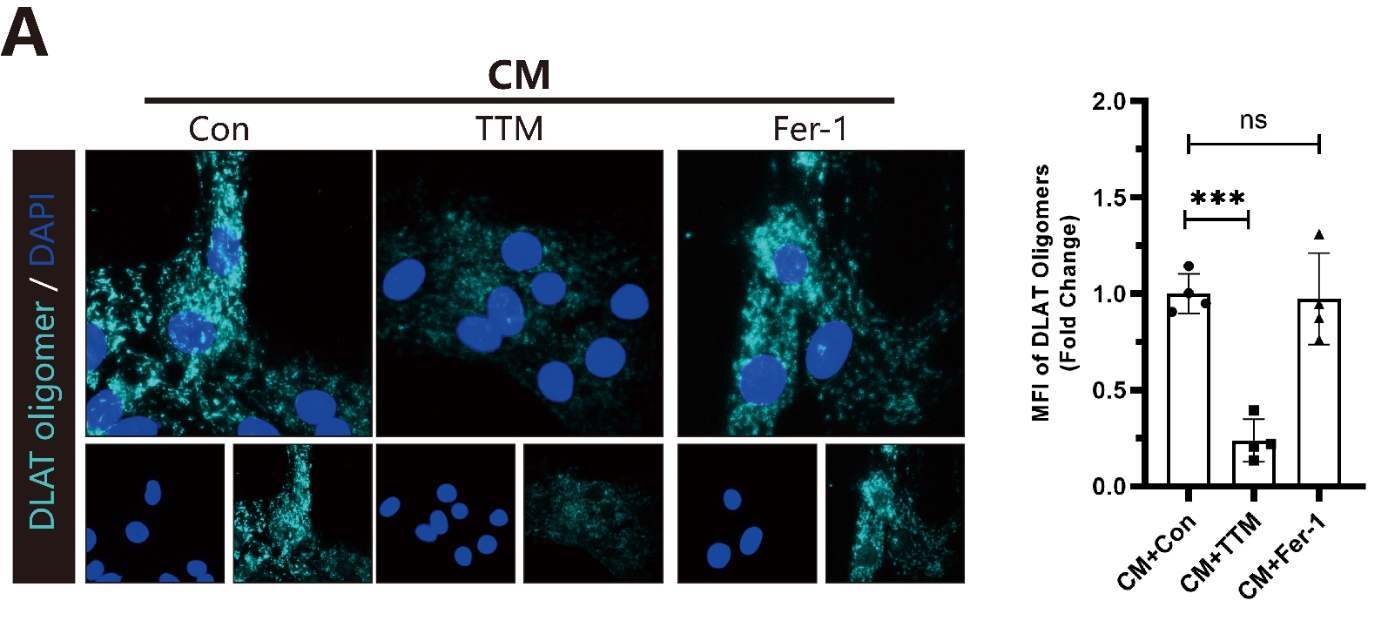


**Supplementary Figure S7. Treatment with Tetrathiomolybdate(TTM) attenuated the aggregation of DLAT in primary rat VSMCs.** Rat VSMCs were treated with TTM or Fer-1 in the presence of CM for 7 days (n = 4). **(A)** Representative images of DLAT oligomer immunofluorescence imaging and quantification, Scale bar=50μm, n=4 per group. Con, Control; CM, calcifying medium; TTM, Tetrathiomolybdate; Fer-1, Ferrostatin-1. ***P < 0.001.


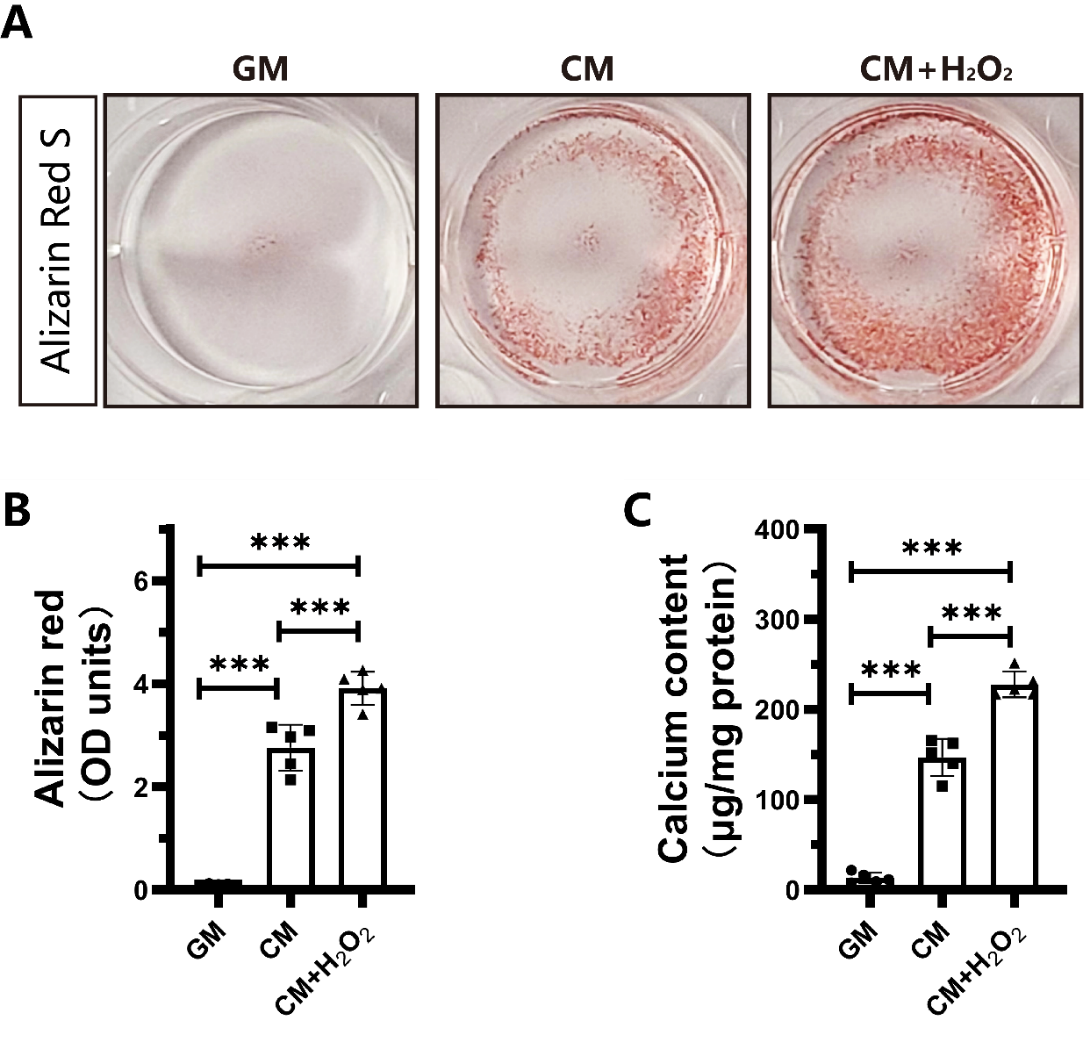


**Supplementary Figure S8. Stimulation of H_2_O_2_ promoted CM-induced calcification in primary rat VSMCs.** Rat VSMCs were treated with GM, CM or CM+H_2_O_2_ for 7 days. (n = 5) (A, B) Alizarin red staining (A) with quantitative analysis (B) was used to assess mineral deposition in primary rat VSMCs. (C) Calcium content was measured by calcium content assay. GM, growth medium; CM, calcifying medium; H2O2, hydrogen peroxide. ***P < 0.001.
